# Lung squamous cell carcinoma and lung adenocarcinoma differential gene expression regulation through pathways of Notch, Hedgehog, Wnt, and ErbB signalling

**DOI:** 10.1038/s41598-020-77284-8

**Published:** 2020-12-03

**Authors:** Dorota Anusewicz, Magdalena Orzechowska, Andrzej K. Bednarek

**Affiliations:** Department of Molecular Carcinogenesis, Medical University of Lodz, 90-752 Lodz, Poland

**Keywords:** Cancer, Cell biology, Oncology

## Abstract

Lung malignancies comprise lethal and aggressive tumours that remain the leading cancer-related death cause worldwide. Regarding histological classification, lung squamous cell carcinoma (LUSC) and adenocarcinoma (LUAD) account for the majority of cases. Surgical resection and various combinations of chemo- and radiation therapies are the golden standards in the treatment of lung cancers, although the five-year survival rate remains very poor. Notch, Hedgehog, Wnt and Erbb signalling are evolutionarily conserved pathways regulating pivotal cellular processes such as differentiation, proliferation, and angiogenesis during embryogenesis and post-natal life. However, to date, there is no study comprehensively revealing signalling networks of these four pathways in LUSC and LUAD. Therefore, the aim of the present study was the investigation profiles of downstream target genes of pathways that differ between LUSC and LUAD biology. Our results showed a few co-expression modules, identified through weighted gene co-expression network analysis (WGCNA), which significantly differentiated downstream signaling of Notch, ErbB, Hedgehog, and Wnt in LUSC and LUAD. Among co-expressed genes essential regulators of the cell cycle, DNA damage response, apoptosis, and proliferation have been found. Most of them were upregulated in LUSC compared to LUAD. In conclusion, identified downstream networks revealed distinct biological mechanisms underlying cancer development and progression in LUSC and LUAD that may diversify the clinical outcome of the disease.

## Introduction

Lung carcinomas remain one of the most aggressive malignancies characterized by the highest mortality rate among men and women worldwide^1,2^. Regarding histological classification lung squamous cell carcinoma (LUSC) and adenocarcinoma (LUAD) account for the majority of lung tumours in non-small cell carcinomas (NSCLCs). In general, the NSCLCs are treated with surgery, which remains the key treatment option, accompanied by various modalities of chemotherapy and radiation. Nevertheless, the five – year survival rate is very poor^3^ and patients experience early events of relapse, metastasis and death^4^.

Despite that both LUAD and LUSC, belong to the family of NSCLCs, they seem very distinct in terms of prognosis as well as the composition of gene expression and signalling pathways profiles. Importantly, more and more often they are being considered as separate clinical entities^5^. LUAD comprises about 40% of all lung cancer cases. In the majority of patients, LUAD mostly affects non-smokers but is also observed among smokers. Usually, the tumour is located more peripherally and grows slower than the other types, although it tends to form metastasis at the early stages of the disease. LUSC, in turn, is the second most common lung malignancy among tobacco smokers. Its pathogenesis is strongly associated with airway lesions that arise with smoking and is mostly located in the central parts of the lung. LUSC is also regarded as a very heterogeneous entity, among which two major subtypes may be distinguished such as basaloid and non-basaloid tumours^6^. Interestingly, lung cancer shows one of the most diverse genetic landscape harbouring numbers of mutations and copy number alterations. By its nature, LUAD bears numerous rearrangements referring to tyrosine kinase receptors (RTKs) such as *ALK*, *ROS1* and *RET*, and mutations that affect known oncogenes (*KRAS*, *EGFR*), which in contrast are very rare or absent in LUSC. These aberrancies tend to affect corresponding signalling pathways and cause global deregulation as they are closely interconnected by cross – talk of their members^7^.

Among all signalling pathways there are few major developmental mechanisms such as Notch, Wnt, Hedgehog (Hh), and ErbB that are mainly indicated in the cancer models of Hanahan & Weinberg^8,9^ as well as Vogelstein et al.^10^ as superior drivers of the carcinogenesis. Additionally, they have been shown strongly involved in lung organogenesis. Notch signalling is evolutionarily conserved pathway determining cell fate during embryogenesis and postnatal life that regulates many cellular processes such as proliferation, differentiation and epithelial–to–mesenchymal transition (EMT), which deregulation may promote carcinogenesis^11^. Specifically for lungs, Notch determines the fate of proximodistal cells at early stages of organ development. Besides, Notch signalling directs later cytodifferentiation of stem and progenitor cells of specific lineages localized within different segments of airways. Notch is also regarded as a key regulator of cellular differentiation in the parenchyma and vascular compartments, thus it may coordinate alveolar epithelium development and capillary formation. Considering the undeniable role of Notch in lung development, as well as given its significance in supervision over cellular proliferation, differentiation and apoptosis, the involvement of this pathway in lung response to injury becomes apparent. Such tight regulation of Notch activation in various lung cell types has also detrimental reflection found in many pathological states such as chronic obstructive pulmonary disease (COPD), pulmonary fibrosis and indeed, lung cancer that arises from inappropriate Notch signalling^12^. Process of lung organogenesis covers likewise epithelial–mesenchymal interactions including cell–cell and cell–matrix interactions necessary for appropriate branching morphogenesis that are in major part dependent on Wnt/β–catenin signalling. Thus, imbalanced location and duration of β–catenin signalling may significantly affect differentiation of the epithelium and the mesenchyme^13,14^. Hh is another developmental pathway appearing to maintain stem cells and response to injuries during adulthood. In lungs, its ligands were found during the formation of the tracheobronchial tree. In addition, Hh participates in the regulatory loop through cooperation with Notch and Wnt promoting differentiation of airway epithelial progenitors to form neuro – and non – neuroendocrine lung components^15^. ErbB pathway, often identified as EGFR—associated pathway transduces signals through the family of four RTKs comprising *Her1/ErbB-1* (*EGFR*, first discovered member) and *Her2—Her4/ ErbB-2—4*, and was identified as a key regulator of lung maturation as well as maintenance of physiological respiratory functions. In humans, lung alveoli are lined by two types of pneumocytes: type I (95% of the alveolar surface) and type II. *EGFR* and its corresponding ligand *EGF* are expressed in type II pneumocytes that secrete pulmonary surfactant. Activation of *EGFR* was likewise specified to modulate the expression of *MUC5A*, the major component of airway mucus, in response to reactive oxygen species (ROS). Moreover, mucin secretion itself increases with *EGFR* activation. Other studies showed that delivery of anti – EGF antisense oligodeoxynucleotides in vivo caused numerous defects in type II epithelial cells and reduced branching morphogenesis in embryos, hence highlighting the important role of proper ErbB signalling in developing lungs. Remaining members of ErbB family with their ligands (*ErbB–2 – 4* and *TGF–α, HB – EGF*, epiregulin and neuregulins) may be found in type I pneumocytes and therein involved in differentiation and developmental processes as well as damage repair through promoting cellular growth^16^. Regarding the prominent role of the pathways discussed in many developmental processes, moreover, in a lung-specific manner, any deregulation during adult homeostasis can lead to various events ultimately leading to the formation of neoplasia. Noteworthy, to date, no study comprehensively describing Notch, Wnt, Hh and ErbB in lung carcinomas in terms of functional cross-talk between their downstream effectors. Moreover, none of them has been correlated with lung cancer subtype, especially at the transcriptome level. Both LUSC and LUAD are members of NSCLC group, although recently they are more often being considered as distinct clinical entities. Therefore, our study aimed to differentiate LUSC and LUAD subtypes at the molecular level and thus reveal functional networks arising from aberrant Notch, Wnt, Hh and ErbB signalling accompanied by correlation with clinical characteristics.

## Results

### Preliminary analysis on pathway alterations in lung cancer—gene set enrichment analysis

**Figure 1:**
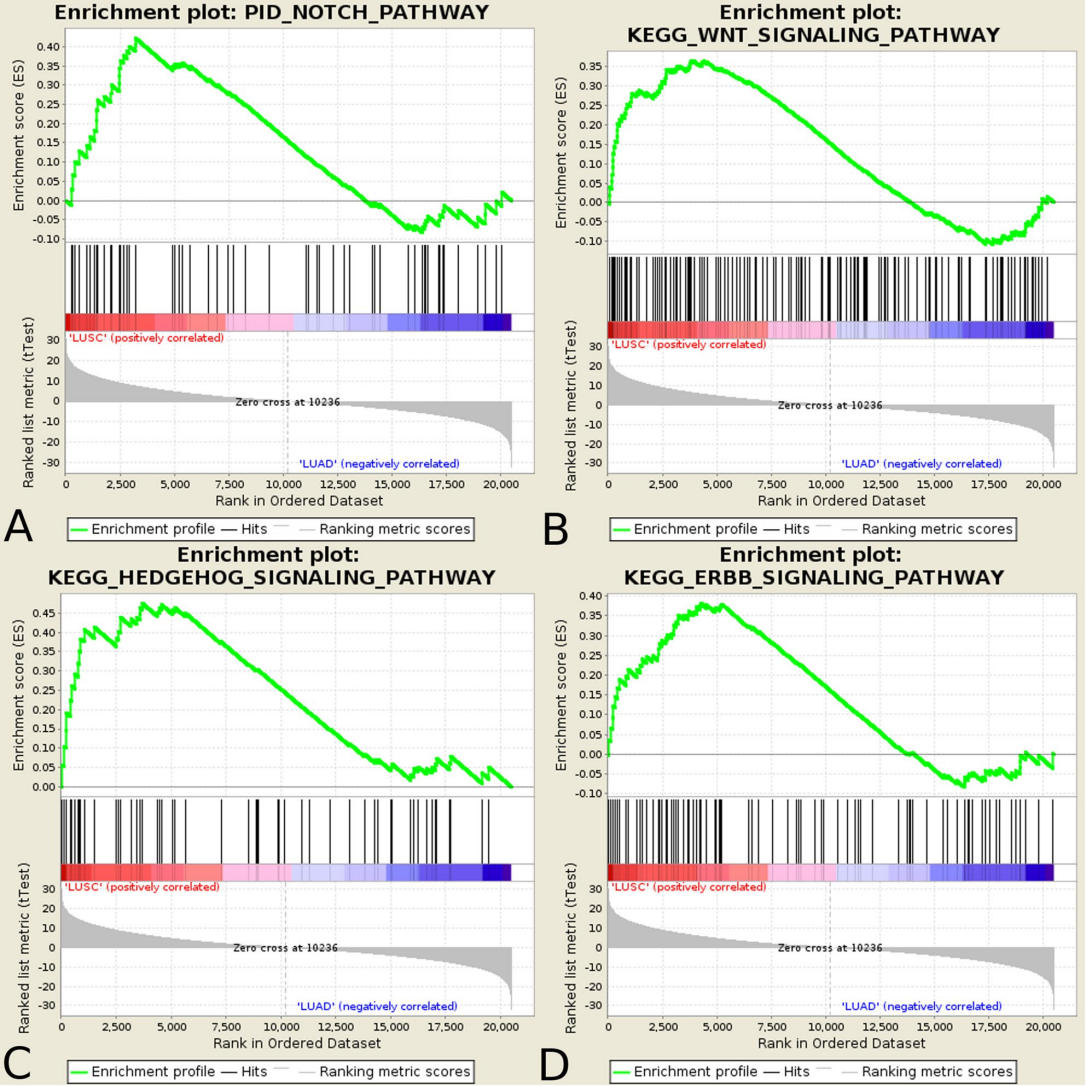
Signalling pathways enriched in lung squamous cell carcinoma and lung adenocarcinoma. Enrichment plots present (**A**) Notch pathway, (**B**) Wnt pathway, (**C**) Hh pathway, and (**D**) ErbB pathway.

Gene Set Enrichment Analysis (GSEA) was performed as preliminary analysis to examine the differentiation in superior signalling pathways between two subtypes of lung carcinoma – LUAD and LUSC. All these highly conserved signal transduction pathways involved in development and tissue homeostasis were enriched in LUSC vs LUAD: PID_NOTCH_PATHWAY (FDR = 0.153, Normalize Enrichment Score (NES) = 1.60), KEGG_WNT_SIGNALING_PATHWAY (FDR = 0.173, NES = 1.56), KEGG_HEDGEHOG_SIGNALING_PATHWAY (FDR = 0.101, NES = 1.69) and KEGG_ERBB_SIGNALING_PATHWAY (FDR = 0.180, NES = 1.54) (Fig. 1).

### Downstream effects of aberrant signalling in LUSC and LUAD through Notch, Hh, ErbB and Wnt

According to GSEA results, we decided to focus on downstream effects of signalling through Notch, Hh, ErbB and Wnt pathways, therefore subsequent analyses considered alterations in target genes of pathway-specific transcription factors, separately for each of the pathways.

### Alterations in signalling networks associated with Notch, Hh, ErbB and Wnt downstream effects in LUSC and LUAD—Weighted Gene Co-expression Network Analysis (WGCNA)

We investigated major biological differences between LUSC and LUAD in signalling networks downstream to Notch, Hh, ErbB and Wnt via WGCNA to find modules of genes that shared common expression profiles among pathway-specific transcription factor’s targets. We analysed The Cancer Genome Atlas (TCGA) data of gene expression of 499 LUSC and 515 LUAD patients. Based on WGCNA pipeline, we identified a total of 9, 12, 9 and 11 distinct co-expression modules for Notch, Hh, Wnt and ErbB pathway, respectively. In all cases, uncorrelated genes were assigned to the grey module and were excluded from the subsequent investigations.

**Figure 2:**
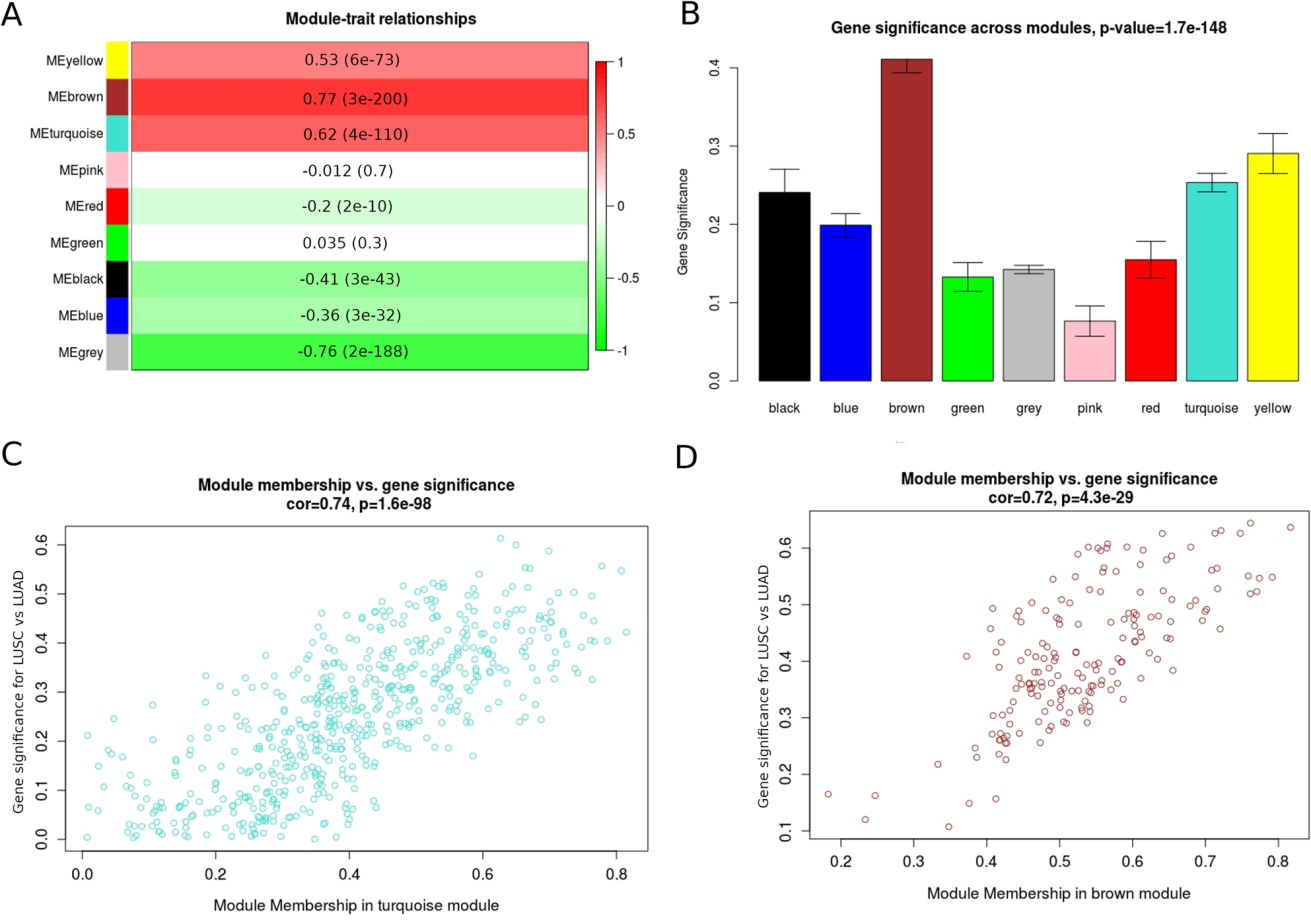
Differential expression of Notch downstream targets vastly characterizing lung cancer subtypes within turquoise and brown modules. These groups were mostly correlated with trait of interest (**A**,**B**) and showed the highest importance of genes within the particular module (**C**,**D**).

**Figure 3:**
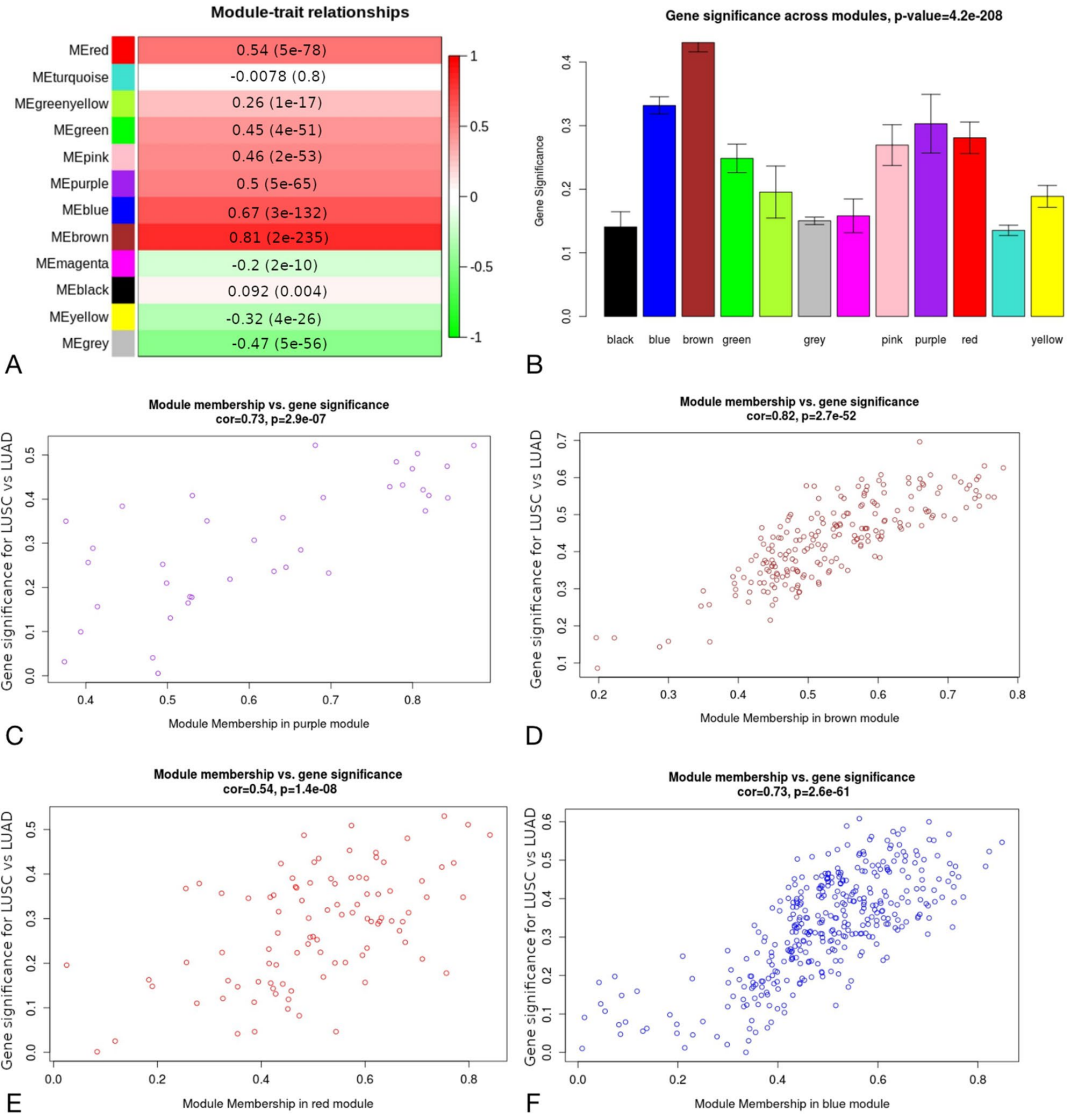
Differential expression of Hh downstream targets vastly characterizing lung cancer subtypes within purple, brown, red and blue modules. These groups were mostly correlated with trait of interest (**A**,**B**) and showed the highest importance of genes within the particular module (**C**–**F**).

**Figure 4:**
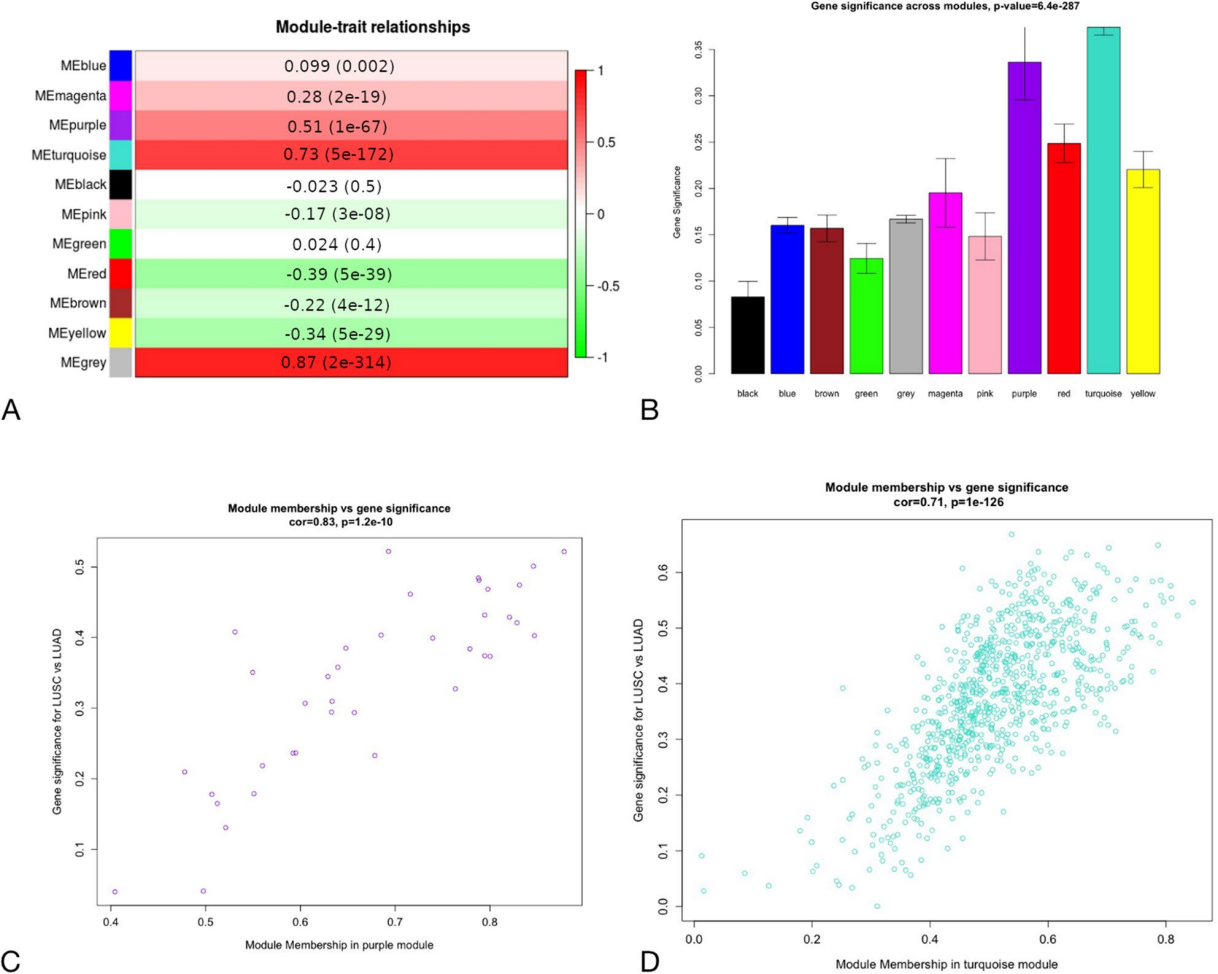
Differential expression of ErbB downstream targets vastly characterizing lung cancer subtypes within purple and brown modules. These groups were mostly correlated with trait of interest (**A**,**B**) and showed the highest importance of genes within the particular module (**C**,**D**).

**Figure 5:**
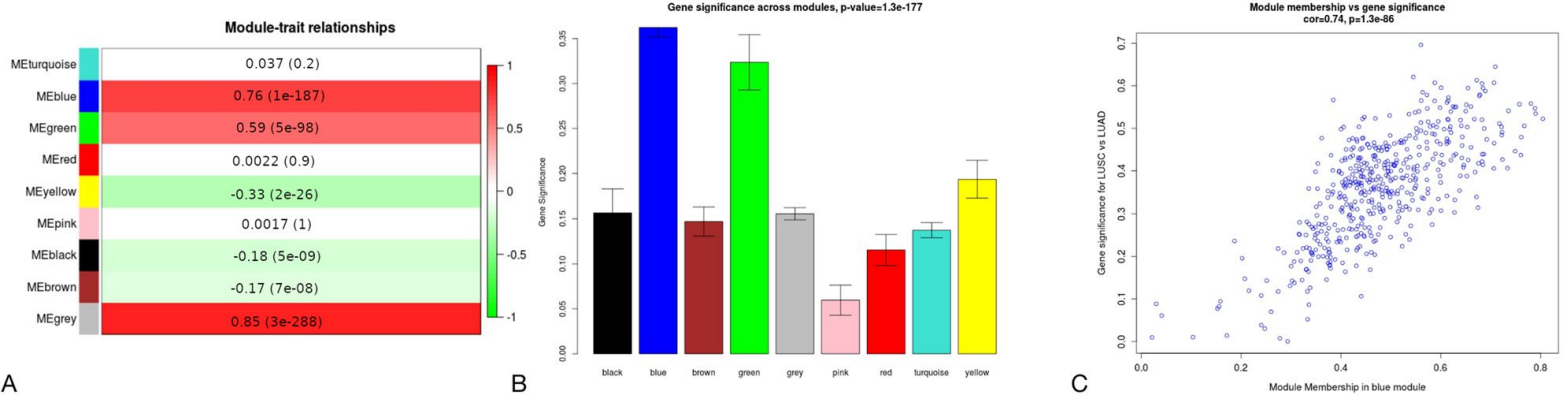
Differential expression of Wnt downstream targets vastly characterizing lung cancer subtypes within blue module. This group was mostly correlated with trait of interest (**A**,**B**) and showed the highest importance of genes within the blue module (**C**).

To understand the biological characteristic of genes from the modules, we performed the correlation analysis between module eigengene (ME) considered as the most representative gene expression profile (the first principle component) of the module and LUSC/LUAD phenotype. According to the module-trait relationship analysis in targets of Notch pathway (Fig. 2A), genes clustered in brown (r = 0.77, p-value = 3e−200), turquoise (r = 0.62, p-value = 4e−110) and yellow (r = 0.53, p-value = 6e−73) modules showed the strongest positive correlation with phenotype. Regarding Hh targets 4 modules were significantly associated with LUSC/LUAD type (Fig. 3A): brown (r = 0.81, p-value = 2e−235), blue (r = 0.67, p-value = 3e−132), red (r = 0.54, p-value = 5e−78) and purple (r = 0.5, p-value = 5e−65). Purple and turquoise modules (r = 0.51, p = 1e−67; r = 0.73, p = 5e−172, respectively) were of the strongest positive correlation with LUSC/LUAD phenotype among ErbB targets (Fig. 4A) and blue and green modules (r = 0.76, p = 1e−187; r = 0.59, p = 5e−98, respectively) among Wnt pathway effectors (Fig. 5A). These MEs were also the most promising as exhibiting the highest gene significance across all modules (Figs. 2, 3, 4, 5, B section).

For each gene in a module, we performed an analysis of the correlation between module membership (MM) and Gene Significance (GS), separately for each module. MM, which is defined as a correlation of gene expression profile with ME (the first principal component of a module), showed the importance of a particular gene across the module. As demonstrated in the scatter plots, among Notch pathway targets MM were highly associated with GS in brown and turquoise modules (brown: cor = 0.72, p = 4.3e−29; turquoise: cor = 0.74, p = 1.6e−98) (Fig. 2C and 2D) and the significant correlation was reported regarding Hh pathway targets for brown (cor = 0.82, p2.7e−52), blue (cor = 0.73, p = 2.6e−61), purple (cor = 0.73, p = 2.9e−07) and red (cor = 0.54, p = 1.4e−08) modules (Fig. 3C–F). We have also found significant associations between MM and GS in blue module in Wnt pathway targets (cor = 0.74, p = 1.3e−86; Fig. 5C) as well as purple (cor = 0.83, p = 1.2e−10) and turquoise (cor = 0.71, p = 1e−126) modules across ErbB pathway effectors (Fig. 4C, D). This analysis highlighted the essential character of particular elements among the modules that tended to show strong association with lung cancer subtype, i.e. LUSC and LUAD.

**Figure 6:**
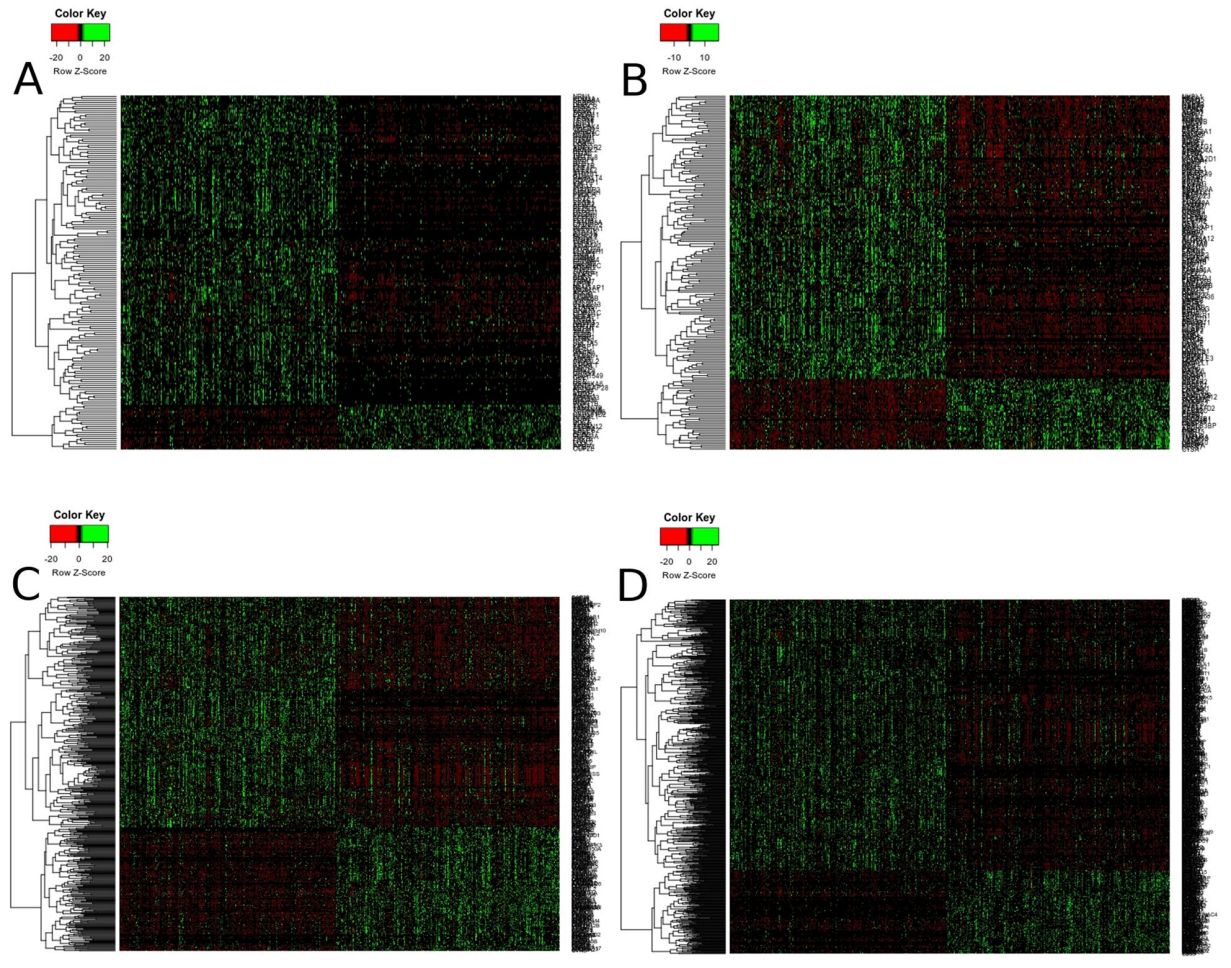
Molecular profiles of (**A**) Notch (brown module), (**B**) Hh (brown module), (**C**) Wnt (blue module), (**D**) ErbB (turquoise module) pathway downstream targets that differentiate lung squamous cell carcinoma from lung adenocarcinoma.

Finally, heatmaps generated for modules of high relevance (brown, turquoise, yellow for Notch targets, brown, blue, purple, red for Hh targets, blue and green for Wnt targets, purple and turquoise for ErbB targets) reflected differential biology of LUAD and LUSC regarding the expression of genes therein (Fig. 6 and Supplementary Figs. 1–4).

### Functional annotation and enrichment analysis of WGCNA modules in Notch, Hedgehog, Wnt and ErbB pathway downstream targets

We summarized the findings of WGCNA with functional annotation of the modules to provide an understanding of biological mechanisms associated with the genes clustered in modules: blue, green of Wnt pathway targets, turquoise, purple of ErbB pathway targets, brown, turquoise, yellow of Notch pathway targets and brown, blue, purple and red of Hh pathway targets. We chose two main categories—KEGG canonical pathways and GO biological processes, both derived from MSigDB. Majority of genes across modules were associated with proliferative and repair processes such as cell cycle, cytoskeleton organization and biogenesis, base excision repair, mismatch repair, MAPK signalling, response to stress as well as developmental processes such as intracellular transport and multicellular organismal development. A detailed description of major findings is shown in Supplementary Table 1.

### Dimensional characteristics of LUSC and LUAD patients—Multiple Factor Analysis (MFA)

**Figure 7:**
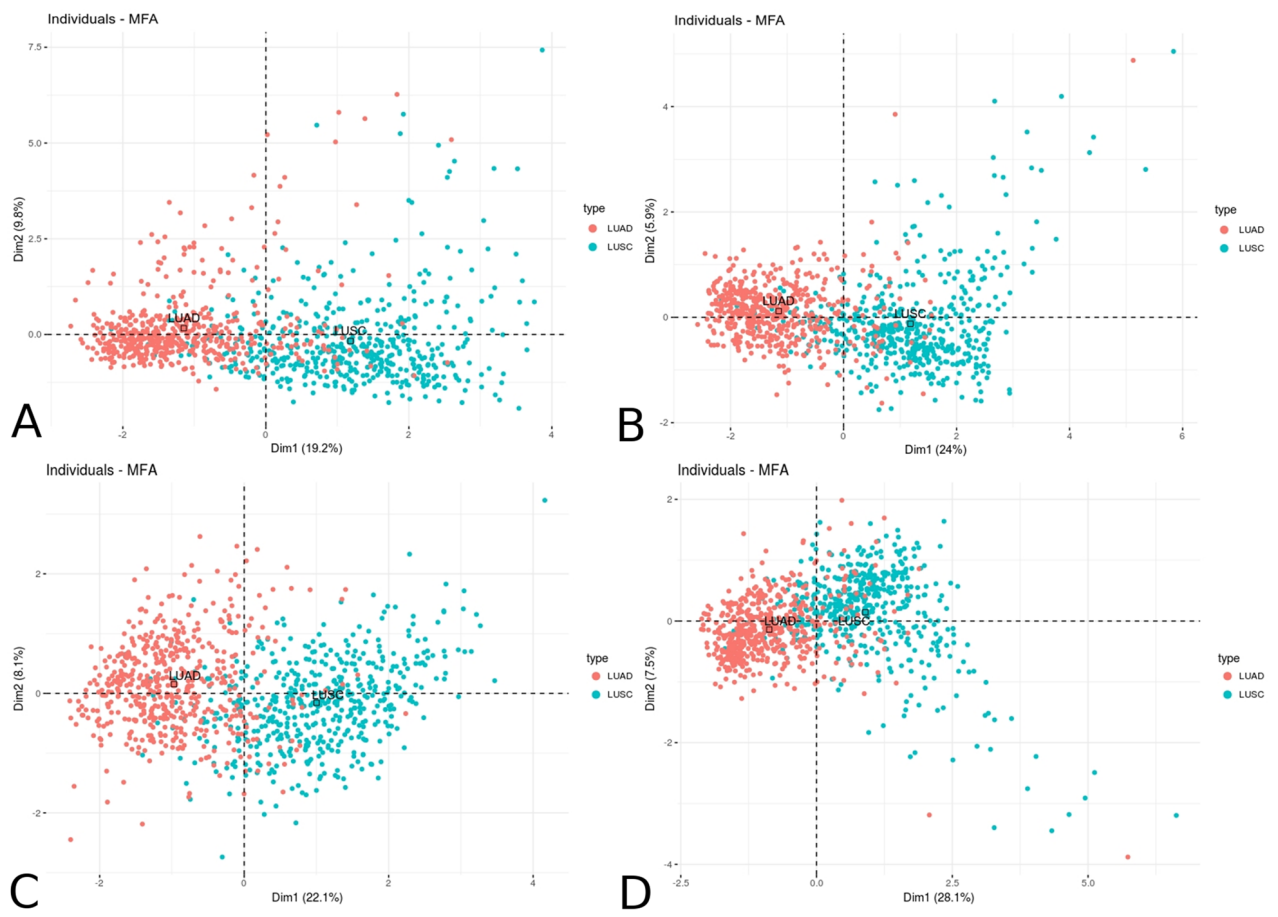
Dimensional partitioning of lung squamous cell carcinoma and lung adenocarcinoma patients according to the resultant expression of the most significant WGCNA modules of (**A**) Notch, (**B**) Hh, (**C**) Wnt and (**D**) ErbB pathway effectors.

To confirm the relationship between the groups of variables (expression of downstream effectors chosen from the highly significant modules and lung cancer subtypes) describing the individuals (patients) concerning their clinical characteristics, we applied Multiple Factor Analysis (MFA). As expected, we found significant partitioning of patients across the first dimension for LUSC/LUAD phenotype with 19.24% variance among Notch effectors, 24.02% variance among Hh effectors, 22.12% variance among Wnt effectors and 28.1% variance among ErbB effectors (Fig. 7).

### Association of the gene expression with patients survival

Additionally, to identify genes that may be of diagnostic-prognostic or therapeutic importance, we analyzed the relationship between the most interesting target genes among the modules with clinical outcomes such as overall survival (OS) and disease-free survival (DFS). Kaplan–Meier survival analysis of the groups of LUSC and LUAD patients was performed using algorithm determining the optimal cutpoint splitting patients into subgroups of more/less favorable outcomes. Of the results, we focused on the genes that proved to have a significant but distinct impact on OS either DFS in both LUAD and LUSC. Regarding OS, we identified genes such as *CDC25A, CDK2, E2F8, KIF11, KIF2A, KIF4A, MAPK8, MCM5, MCM6, MYC, PARP1*, and *PIK3CA* that significantly differed in the patients' outcome between LUSC and LUAD. Additionally, we identified unique genes significantly altering the prognosis only in one of the tumors such as *CDK16* and *MSH2* for LUSC and *BRCA1, BRCA2, CCNB1, CDKN3, E2F1, E2F2, KIF14, KIF23, MAPK6, MCM2, MCM4, MCM8, MCM10*, and *RAD51* for LUAD. Regarding DFS, we identified the unique genes such as *BRCA1, BRCA2, KIF14, MCM5, MCM8, MSH2, PARP1*, and *TP63* for LUSC and *E2F2, MAPK6, MAPK8, MCM2* for LUAD (statistics data are shown in Tables 1 and 2).

**Table 1:** Prognostic effect of chosen target genes on OS in lung subtypes.

GENE	LUSC					LUAD				
	HR	P-value	Cutpoint	< Cutpoint	> Cutpoint	HR	P-value	Cutpoint	< Cutpoint	> Cutpoint
*CDC25A*	0.608	0.00388	295.5	317 (64%)	182 (36%)	1.71	0.00322	131.9	365 (71%)	150 (29%)
*CDK2*	0.613	0.0302	1530	425 (85%)	74 (15%)	1.54	0.0229	617.8	243 (47%)	272 (53%)
*E2F8*	2.11	0.00841	71.46	61 (12%)	438 (88%)	1.54	0.0395	255.8	425 (83%)	90 (17%)
*KIF11*	0.652	0.0102	1116	296 (59%)	203 (41%)	1.92	0.00132	353.7	201 (39%)	314 (61%)
*KIF2A*	1.52	0.0234	500.7	173 (35%)	326 (65%)	1.7	0.0156	375.3	104 (20%)	411 (80%)
*KIF4A*	0.713	0.0467	831.2	312 (63%)	187 (37%)	1.68	0.00457	456.3	336 (65%)	179 (35%)
*MAPK8*	1.82	0.0114	193.3	112 (22%)	387 (65%)	1.45	0.0483	215.8	236 (46%)	279 (54%)
*MCM5*	1.41	0.0489	1891	161 (32%)	338 (68%)	1.78	0.00128	1251	319 (62%)	196 (38%)
*MCM6*	0.624	0.0121	2080	369 (74%)	130 (26%)	1.64	0.00864	1048	244 (47%)	271 (53%)
*MYC*	1.45	0.0407	2308	167 (33%)	332 (67%)	1.91	0.000687	2026	395 (77%)	120 (23%)
*PARP1*	0.0104	0.0104	4448	190 (38%)	309 (62%)	2.82	0.000176	7323	482 (94%)	33 (6%)
*PIK3CA*	0.0487	0.0487	649	168 (34%)	331 (66%)	2.18	1.55E-05	429.2	333 (65%)	182 (35%)
*CDK16*	0.56	0.011	1851	52 (10%)	447 (90%)					
*MSH2*	0.626	0.0113	1206	340 (68%)	159 (32%)					
*BRCA1*						1.69	0.0106	199.3	184 (36%)	331 (64%)
*BRCA2*						2.06	0.00054	200.3	432 (84%)	83 (16%)
*CCNB1*						1.94	0.000392	680	261 (51%)	254 (49%)
*CDKN3*						1.64	0.00641	144.6	292 (57%)	223 (43%)
*E2F1*						1.62	0.0263	268.1	176 (34%)	339 (66%)
*E2F2*						1.65	0.0175	84.59	167 (32%)	348 (68%)
*KIF14*						1.87	0.00051	195.5	307 (60%)	208 (40%)
*KIF23*						1.82	0.000855	433.7	334 (65%)	181 (35%)
*MAPK6*						2.22	3.31E-05	1594	405 (79%)	110 (21%)
*MCM10*						1.58	0.0168	108.3	219 (43%)	296 (57%)
*MCM2*						1.52	0.0211	1016	279 (54%)	236 (46%)
*MCM4*						2.1	3.61E-05	1413	303 (59%)	212 (41%)
*MCM8*						1.58	0.0188	177.8	177 (34%)	338 (66%)

**Table 2:** Prognostic effect of chosen target genes on DFS in lung subtypes.

GENE	LUSC					LUAD				
	HR	P-value	Cutpoint	< Cutpoint	> Cutpoint	HR	P-value	Cutpoint	< Cutpoint	> Cutpoint
*BRCA1*	2.54	0.0157	435.6	201 (40%)	298 (60%)					
*BRCA2*	5.24	0.000186	386.4	465 (93%)	34 (7%)					
*KIF14*	2.54	0.0215	258.1	168 (34%)	331 (66%)					
*MCM5*	3.06	0.0264	1729	117 (23%)	382 (77%)					
*MCM8*	2.86	0.000848	708.6	359 (72%)	140 (28%)					
*MSH2*	2.43	0.00718	1579	420 (84%)	79 (16%)					
*PARP1*	3.48	0.000885	8435	461 (92%)	38 (8%)					
*TP63*	0.382	0.0176	13,310	333 (67%)	166 (33%)					
*E2F2*						0.363	0.045	232.2	428 (83%)	87 (17%)
*MAPK6*						0.522	0.033	1067	262 (51%)	253 (49%)
*MAPK8*						0.398	0.0449	315.7	406 (79%)	109 (21%)
*MCM2*						1.58	0.0188	1268	348 (68%)	167 (32%)

## Discussion

Lung cancer is a very complex and heterogeneous disease, categorized into two major types, small cell lung carcinoma (SCLC) and NSCLC. NSCLCs account for approx. 85% of all lung cancers and is associated with high rates of proliferation and metastases as well as poor prognosis for advanced-stage disease^17^. In this study, we focused on LUAD and LUSC among all NSCLCs. LUSC manifests in poorer prognosis and usually arises as a tumour localised in the proximal part of the bronchial tree and is strongly associated with tobacco smoking, whereas LUAD is usually peripherally located and occurs more often among non-smokers^18^. Since both subtypes vary in clinical and histopathological features, their molecular mechanism of carcinogenesis and tumour progression may be diverse.

The development of NGS technology has enabled to study the expression level of many genes simultaneously and understand how the networks and pathways interact with each other. To identify the distinct molecular profiles based on gene expression analysis of LUSC and LUAD, we focused on four evolutionary conserved signalling pathways: Notch, Hh, Wnt and ErbB, which tightly regulate proliferation, differentiation, apoptosis, migration and motility. Previous studies showed that the EGFR/ErbB pathway is involved in LUAD pathology with mutations of EGFR and ERBB2 identified in 11% and 3% of LUAD cases, respectively^19^. Another group reported differential and clinically important subtypes of LUSC based on gene expression profiles^20^. Finally, a recent comparison of LUSC and LUAD showed distinct expression profiles of genes involved in tumour immune response^21,22^. Alternations of Notch, Hh, Wnt and ErbB pathways were repeatedly associated with development and progression of many malignancies. Our GSEA analysis revealed that Notch, Hh, Wnt, and ErbB pathways were significantly overrepresented in LUSC compared to LUAD. Regarding the differential expression of the genes directly involved in these pathways, it seems logical to assume that there are alternations in their downstream target genes, which may influence the molecular and clinical character of LUAD and LUSC. Thereby, identification of their differential signalling in NSCLC may help to understand the biology of lung cancer and lead to new personalized therapies in the future.

By using WGCNA we compared gene expression profiles between subtypes of lung cancer and revealed 11 co-expression modules of Notch, Wnt, Hh and ErbB downstream targets showing a strong positive correlation (cor > 0.5) with either LUSC or LUAD subtype. These modules corresponded to a total of 1965 differentially expressed genes of potential biological relevance associated with gene ontology terms such as cell cycle, cellular differentiation and proliferation, DNA repair, metabolic processes and apoptosis (Supplementary Table 1) that point out the major biological differences between LUSC and LUAD.

Major differences included genes involved in the cell cycle control and some of them have been already known to be involved in tumourigenesis^23,24^. Loss of checkpoint and integrity induced by failures of the cell-cycle machinery, triggering uncontrolled tumour proliferation and leading to malignant transformation. In this context, our analysis has shown that many of the cell cycle genes were elevated in LUSC compared to LUAD. Among them we identified cyclins, cyclin-dependent kinases (CDKs), E2F family transcription factors, kinesin superfamily proteins (KIFs) and minichromosome maintenance proteins (MCMs) (Supplementary Figs. 4; Figs. 3 A, C and D), thus revealing different effects of abolished cell cycle control through Notch, Hh, Wnt and ErbB pathways.

The balance of cellular proliferation and apoptosis plays a pivotal role in the control of tumour growth, which as we found, seems to be driven by the effects of abrogated Notch signalling (Supplementary Table 1). Deregulation of apoptosis is implicated in tumour initiation, progression and drug resistance in many human cancers and is also identified as one of the hallmarks of cancer^8,9^.

Another interesting group of genes are DNA repair genes, which play a key role in genome surveillance and protection. Functional deregulation of DNA repair is one of the common features of highly aggressive human malignancies^25^. There are at least eight distinct DNA repair mechanisms in human cells, amongst which are: base excision repair (BER), nucleotide excision repair (NER), mismatch repair (MMR), non-homologous end-joining (NHEJ), homologous recombination (HR) and Fanconi anaemia pathway (FA)^25–27^. Additionally, *BRCA1* and *BRCA2* expression was shown to differentiate LUSC and LUAD, and their reduced expression was previously reported as associated with hypermethylation of gene promoters in LUAD^28^.

Our analysis has shown that many of the cell cycle genes, including cyclins and CDKs, were elevated in LUSC compared to LUAD. Especially, overexpression of *CDK2* and *CDK16* was shown to cause abnormal regulation of cell cycle and to promote cell proliferation^29^. Our findings are in compliance with previous research that indicated higher expression of *CDKN3* in LUSC than in LUAD^30,31^, whereas overexpression of cyclin B1 (*CCNB1*, a key molecule for G2/M phase) was recently identified as a predictive marker of worse overall survival among LUSC patients^7^. Moreover, high expression of *CCNB1* increased cell differentiation, high proliferative index, vascular invasion and thus increased malignant potential^32,33^. *CDC25A* in turn seemed to possess oncogenic properties and its overexpression was frequently associated with the malignancies and poor prognosis^34,35^.

Another group of genes controlling the cell cycle comprises the family of E2F transcription factors. One of their abundant functions is a regulation of expression of genes essential for the transition from G1 to S phase of cell cycle like *Cdc25a*, cyclin A and cyclin B^36^. Our analysis revealed an upregulated expression of *E2F1* and *E2F2* in LUSC compared to LUAD. Several studies reported that E2F factors likely contribute to lung carcinogenesis^37^. The overexpression of *E2F1* has been associated with the development of NSCLC and indicated worse prognosis^38,39^. *E2F2* expression was predominantly elevated in NSCLC tumours and was also shown to correlate with cell proliferation leading to tumour progression^37^. Park et al. found overexpression of *E2F8* in lung cancer cell lines and in lung cancer tissue samples that was ultimately associated with poorer prognosis. Moreover, in the same study inhibition of *E2F8* suppressed cell proliferation, colony formation and invasion and tumour growth in vitro and in vivo^40^.

Kinesin superfamily is essential for mitosis and meiosis, intracellular transport and cell migration^41^. In our analysis, we demonstrated that several genes of the KIF family are heightened in LUSC. To date, it has been established that overexpression of specific genes such as *KIF4A* and *KIF14* is strongly associated with poorer prognosis of NSCLC cases^42,43^. Significant increase in *KIF2A* expression in NSCLC was additionally associated with lymph node metastasis^44^ as well as LUAD progression^45^. Our study revealed differences in *KIF11*, the upregulation of which was previously reported in LUSC compared to LUAD^46^.

We found that genes of MCM family involved in eukaryotic genome replication were heightened in LUSC vs LUAD (Supplementary Figs. 2–4 and Fig. 6 B, C and D). In the previous reports, MCM genes have been proposed as prognostic biomarkers of proliferation in lung cancer^47–51^. NSCLCs displayed elevated expression of *MCM2* as well as *MCM5* and *MCM6* that were associated with patients’ shorter overall survival. Additionally, the same study reported that higher *MCM5* was significantly correlated with distant metastases^49^. In our research, the expression of all above mentioned MCM genes were higher in LUSC than in LUAD, while only *MCM5* were found to have the same trend in survival. Moreover, higher expression of *MCM4* was associated with non-adenocarcinomas as well as smoking, and we confirmed the above finding demonstrating upregulation of *MCM4* in LUSC^48^.

Cancer cells often show abolished signal transduction that leads to proliferation in response to external signals. Among the most differentiated genes between LUSC and LUAD we found genes involved in frequently deregulated pathways during carcinogenesis, e.g. *MYC, TP63, GSK-3β, PIK3CA, MAP* kinases (Figs. 6 A, B and D and Supplementary Figs. 1, 2 and 4). Amplification of c-myc was shown to play an important role in such processes as metastasis, invasion and resistance to chemotherapy^52,53^. *TP63* gene amplification and corresponding protein overexpression have been so far documented mainly in squamous cell carcinoma and related to the tumour proliferation fraction^54,55^. Glycogen synthase kinase-3 (GSK-3) is a key regulator of numerous signalling pathways during embryogenesis and in metabolic control. We found that *GSK-3β* isoform exhibited higher expression in LUSC patients. Respectively, previous studies have shown that overexpression of the *GSK-3β* in NSCLC patients regulated cell proliferation, tumourigenesis, apoptosis and cell invasiveness and thus was identified as a risk factor of poor prognosis^56^. *PIK3CA* is one of the most frequently mutated genes in human cancers and its somatic mutations have also been reported in lung cancer^57^. Our analysis showed differential expression of *PIK3CA* in NSCLC but there are no reports of *PIK3CA* gene overexpression being associated with either carcinogenesis or progression of lung cancer. However, elevated expression of *PIK3CA* has been reported in various types of cancer including oesophageal squamous cell carcinoma, colorectal and breast cancer where was related to invasiveness, metastasis and poor prognosis^58–60^. *MAPK* has been linked to cell proliferation and transformation. In our study diverged expression levels of *MAPK6* and *MAPK8* were found between LUSC and LUAD, with higher expression in LUSC. *MAPK6* (Supplementary Figs. 2–3, Figs. 6 B and C), also known as *ERK3*, were found to be strongly upregulated in human lung carcinoma and promotes cancer cell invasion^61^. *MAPK8* (*JNK1*) is involved in transduction of extracellular signals such as growth factors or cytokines and also was to be an important contributor to the tumour promoting activity of tobacco smoke in lungs^62^.

Many of the DNA repair genes were expressed differently in LUSC and LUAD, which may suggest that the development and aggressiveness of these tumours dependents on distinct mechanisms. Homologous recombination is an error-free double-strand break (DSB) repair pathway active during the S and G2 phases of the cell cycle due to the necessity of a sister chromatid for use as a homologous template^63^. Among genes involved in the HR, we identified *RAD51, BRCA1* and *BRCA2*. Decreased BRCA expression caused by methylation or mutation has been shown to impair the homologous recombination. In addition, aberrant protein expression as well as low mRNA level of *BRCA1* and *BRCA2*, was significantly associated with promoter hypermethylation of these genes, especially in LUAD patients^28^. These findings are in compliance with our results demonstrated lowered expression of *BRCA1* and *BRCA2* in LUAD compared to LUSC (Fig. 6D). The above suggests that alterations of the key members of the DSB repair pathway are important primarily in the pathogenesis of LUAD. *RAD51* is a protein, which interacts with various tumour suppressor, including *BRCA1*, *BRCA2*, *TP53*. High level of *RAD51* was related to chemo- and radioresistance of lung cancer^64,65^ as well as the enhanced propensity of cancer cells to survive and avoid apoptosis. Significantly shorter survival was also observed among NSCLC patients with higher *RAD51* expression^65^. In turn, *PARP1* and *PARP2* have been described as active players of the DNA damage response, DNA metabolism and chromatin architecture. They are important in BER recognition of single-stranded breaks. Importantly, our analysis has revealed that both *PARP1* and *PARP2* were upregulated in LUSC compared to LUAD. Chen K. et al. reported that *PARP1* is strongly expressed in metastatic NSCLC and facilitates migration and invasion of NSCLC cells. Moreover, the overall survival was significantly lower in *PARP1* high expression group of patients than in *PARP1* low expression group^66^.

MMR is a highly conserved biological mechanism that recognizes and repairs erroneous insertions, deletions and base substitution that have been neglected by the intrinsic proofreading activity of the DNA polymerases^67^. Inactivation of MMR induces a mutator phenotype and causes a predisposition to cancer. Indeed, loss of *MSH2*, one of the key components of the MMR, influences the enhancement of genomic instability. Our analysis showed reduced expression of *MSH2* in LUAD. Previous studies showed in turn that low expression of *MSH2* was positively correlated with decreased overall survival of lung cancer patients due to increased genome instability, a hallmark of MMR-deficient cells^68^. We found that *MSH2* together with *MSH6* have lower expression in LUAD patients than among LUSC.

Analysis of gene ontology (GOs) and pathways identified diverse gene sets, suggesting that these may serve primarily roles in differentiation of LUSC and LUAD pathogenesis. Moreover, we aimed to evaluate if any of the differentially expressed genes had a potential prognostic impact for LUAD or LUSC. The Evaluate Cutpoint analysis led us to discover a few important genes that could differentially determine disease outcome as well as disease free survival in LUAD versus LUSC. Higher expression of *E2F8, MAPK8, MCM5 and MYC* followed the same, negative, prognostic impact on both LUSC and LUAD, however taking into account the number of patients with expression of these genes above cutpoint (in LUSC: 88% for *E2F8*, 78% for *MAPK8, 68*% for *MCM5* and 67% for *MYC*; in LUAD: 17% for *E2F8*, 54% for *MAPK8, 38*% for *MCM5*, 23% for *MYC*), it could be assumed that frequently elevated expression of these genes in patients with LUSC may determine the final outcome. The most interesting from viewpoint of distinguish LUSC from LUAD regarding survival rate are genes with opposite trends such as *CDC25A, CDK2, KIF11, KIF4A, MCM6, PARP1* and *PIK3CA* and genes unique for LUSC or LUAD (Table 1) and these genes could be proposed as potential separate prognostic factor for LUSC and LUAD. All of these genes are favorable for LUAD when their expression is lower than cutpoints, meaning that patients with increased expression of these genes will have poorer overall survival. However, in the vast majority of patients of studied cohort, most of these genes indicated lower expression level than cutpoints which might be related with lower effect of studied signaling pathway.

The inverse correlation of *KIF11* and *KIF4A* with overall survival in LUAD vs LUSC could be related with various regulation of mitosis and cellular transport between both of this subtypes. Abnormal kinesin expression could alter the equal distribution of genetic materials during cell mitosis leading to numerous defects in the daughter cells^69^ thus targeting specific kinesins may create a strategy for differentiation of LUSC and LUAD. Similarly, different trends of overall survival in case of *CDC25A* and *CDK2* could indicate other course of cell cycle associated with disease progression that determines OS. Interestingly, similar level of *PIK3CA* cutpoint expression showed completely opposed effect for patients survival. *PIK3CA* is one of the PIK isoform taking part in signal transduction and is frequently mutated in cancer^70^ however our analysis showed that its expression status might play role in predicting the different therapeutic effect in LUAD and LUSC. Moreover, gene such as *KIF2A,* due to the large number of patients in the cohort with increased (unfavorable) expression of this gene, should be considered as an important potential target in LUAD.

Interestingly, the results for DFS do not correspond in large extent with the results for OS. Our findings suggest that high expression of *BRCA1, BRCA2, KIF14, MCM5, MCM8, MSH2, PARP1* were unfavorable indicator of DFS in LUSC, whereas low expression of *E2F2, MAPK6, MAPK8* and high expression of *MCM2* were unfavorable indicator of DFS in LUAD (Table 2). This results suggest completely different mechanism of recurrence in these two lung cancer subtypes.

To summarize, the great potential of biomarker we proposed for being a prognostic factor is based on their biological background, primarily allegiance to biological processes frequently altered in cancer. However, qualifying patients to the favorable or unfavorable prognosis group based only on the determination of the cutpoint for a given gene is insufficient, as optimal cutpoints are usually dataset dependent thus could be unlikely to be the same in other studies. Moreover as single-variable relations do not take into account the values of all the other predictors, therefore further analyzes as well as in vitro research that could confirm our statements would be needed.

Compiling all data, it becomes evident that extremely significant differences exist between gene expression profiles of LUSC and LUAD concerning distinct downstream outcomes of various signalling pathways such as Notch, Wnt, Hh and ErbB. Overexpression of well-known oncogenes like *PIK3CA, MYC, RAD51* as well as genes involved in cell cycle and DNA repair, suggest that LUSC might have enhanced aggressiveness and migratory potential positively affects its predisposition to metastasis. Worth noting, the fact that LUSC is more common among “smokers” is not irrelevant for deregulation of these genes. The exposure to the DNA damaging factors contained in tobacco could be one of the causes of differences found between LUSC and LUAD. On the other hand, lower expression of DNA repair key players like *BRCA1, BRCA2, MSH2* and *MSH6* in LUAD could suggest that strategies of DNA repair in LUSC are very distinct from those in LUAD. The analyzed ontologies clearly differentiate LUSC from LUAD in terms of the expression, especially expression of particular genes. More importantly, further analysis have identified sets of genes that differently affect patients overall survival and disease free survival depending on tumor subtype and thus we conclude that they should be taken into account as separate set of potential prognostic markers for LUAD and LUSC. Our research provides direction for clinical treatment and molecular mechanism insight of differentation between LUAD and LUSC that may help developing and assessing novel diagnostic and prognostic procedures for lung cancer.

The limitation of the present study might be the fact that there is no perfect way to validate the results. The publicly available resources offering RNAseq data are very limited; due to that reason to cross-validate and corroborate our findings, we have retrieved gene expression profiles from microarray platforms via GEO repository. Nevertheless, we succeeded to confirm the primary results to a large extent. On average, about 50% of genes that overlapped between TCGA and GEO-derived WGCNA modules showed the same trend in expression as well as partitioned patients according to LUSC/LUAD phenotype in PCA. It should be also stated that the observable remaining discrepancies between primary and cross-validation studies may have arisen for several reasons. Firstly, the normalization methods that apply to RNAseq and microarrays are very distinct regarding both, technology and data processing. Secondly, according to Tian F et al. RNAseq compared to microarray technology shows in general only 67–68% average reproducibility^71^. Finally, the cohorts itself may be biased or potential batch effect exists.

Summarizing, subtyping of LUAD and LUSC based on gene expression provides valuable information regarding differential biological mechanisms of cancer development and invasiveness reflected in clinical features of NSCLC tumours that stem from abrogated evolutionary signalling and its downstream outcomes.

## Materials and methods

We obtained the RNA-Seq data of 515 LUAD and 501 LUSC cancer samples (RNAseq, level 3 RNASeqV2, RSEM normalized) and clinical data of the 522 LUAD and 501 LUSC patients from TCGA, downloaded from http://gdac.broadinstitute.org/ (data status of Jan 28^th^, 2016). The present study included the analysis of the data that have been collected and processed by The Cancer Genome Atlas Research Network, therefore no approval of the institutional committee was required. The methods of biospecimen procurement, RNA isolation and RNA sequencing were previously described by The Cancer Genome Atlas Research Network^28,72^. All experimental protocols were approved by a named institutional or licensing committee and the informed consent from all subjects or, if subjects were under 18, from a parent or legal guardian was obtained, as described therein. All methods were carried out in accordance with relevant guidelines and regulations.

The TCGA RNAseq data were combined with the patients’ clinical outcome. Patients with missing any clinical or expression value were excluded from further analysis. Finally, we qualified a total of 515 LUAD and 499 LUSC samples. The summary of clinical characteristics of the both cohorts is shown in Supplementary Table 5.

To explore significant differences between patients with LUAD and LUSC we performed GSEA^73^. Enrichment analysis was applied to 20,502 genes in terms of the Canonical Pathway database. Enrichment was subjected to GSEA by applying t-test with a weighted scoring scheme, default 1000 permutation and permutation type regarding the phenotype, using the default significance threshold of FDR < 0.25. To achieve the reproducibility of results we used the precise number in random seed parameter, which was 779,948,241.

According to GSEA results, we decided to focus on downstream target genes of four pathways: Notch, Hedgehog, Wnt and ErbB. Through the Gene Transcription Regulation Database (GTRD), available online at http://gtrd.biouml.org/^74,75^, we made a list of targets of pathway-specific transcription factors, separately for each of the aforementioned pathways. We listed a total of 2949 downstream target genes of *HES1, HES2, HES4, HES7, HEY1, HEY2* and *HEYL*, 2981 targets genes of *GLI1, GLI2* and *GLI3*, 2571 target genes of *LEF1, TCF3* and *TCF4* and 5912 target genes of *Elk-1, c-Myc, c-Jun, c-Fos, STAT5A, STAT5B, FOXO1* for Notch, Hedgehog, Wnt and ErbB pathways, respectively.

Weighted gene co-expression networks were built using the WGCNA package in the R environment^76^. A more detailed description can be found at https://labs.genetics.ucla.edu/horvath/CoexpressionNetwork/Rpackages/WGCNA/. Briefly, pairwise Pearson’s correlation matrix of expression was calculated and then transformed into an adjacency matrix. With function pickSoftThreshold we utilized soft-thresholding approach (β = 6 for Notch, Hh, Wnt pathway and β = 8 for Erbb pathway), to ensure a scale-free topology of the network with scale-free topology index (R2) > 0.80. Then, we used the adjacency matrix to construct the topological overlap matrix (TOM), which corresponds to the overlap between pairs of interconnected genes. TOM was used to produce hierarchical clustering tree of genes, by hclust R function and “average” as a method. Genes sharing common expression profiles were clustered into modules by dynamicTreeCut algorithm with minModuleSize = 30 and other parameters set to default. To identify modules that were significantly correlated with the trait of interest – a subtype of lung cancer, we calculated the correlation between ME and clinical trait. ME was considered as the first principal component of each gene module. For each module, we also defined module membership as a correlation between ME and gene expression.

Afterwards, GS was defined as the log10 transformation of the p-value in the linear regression between gene expression and external trait. Besides, module significance (MS) was defined as the average GS for all the genes in a module.

The annotation of the gene ontology terms within WGCNA modules was performed through WGCNA-dedicated R packages such as anRichment and anRichmentMethods involving MSigDB repositories, i.e. C2 KEGG canonical pathways and C5 GO Biological Processes.

Furthermore, we performed spatial grouping of lung cancer patients through MFA according to various variables to determine the relevance of subtype (LUAD vs LUSC) and further associations with combined expression of genes derived from the most prominent WGCNA modules. Each module was treated as a separate group and the analysis was applied for each pathway separately. The MFA was applied using packages: FactoMineR and factoextra^77^ within the R environment.

We performed a survival analysis using Evaluate Cutpoints application for R environment^78^. Overall Survival (OS) and Disease Free Survival (DFS) was estimated by determination of optimal cutpoint splitting patients into two subgroups of favorable and unfavorable prognosis regarding expression of particular target genes as biomarker. In our analysis we used cutp algorithms of cutpoint determination in correlation with survival time and clinical outcome according to the following clinical parameters: “patient.person_neoplasm_cancer_status” and “patient.vital_status” as event indicator and “patient.days_to_last_followup” and “patient.days_to_death” as time of observation for DFS and OS, respectively.

To cross-validate data reliability and reproducibility, we extracted the microarray data of lung cancer patients from the GEO database (https://www.ncbi.nlm.nih.gov/geo/). The chosen series, GSE4573 and GSE12667, were based on the Affymetrix Human Genome U133A Array and Affymetrix Human Genome U133A Plus 2.0 Array platforms, respectively. The GSE4573 dataset submitted by Raponi et al. included 130 squamous cell lung carcinoma samples and 22,284 probesets^77^, whereas GSE12667 submitted by Ding et al. included 75 lung adenocarcinomas samples and 54,676 probesets^78^. For validation purposes, we extracted 22,277 probesets that overlapped between the Raponi’s and Ding’s projects and conducted WGCNA analogously to the primary analysis with soft thresholding power of β = 20. Several modules of initially identified through dynamicTreeCut algorithm tended to share very similar expression profiles, therefore we decided to merge them at height cut of 0.05, which corresponded to eigengenes correlation of 0.95. The annotation of ontological terms regarding C2 KEGG canonical pathways and C5 GO Biological Processes was additionally performed for all relevant modules of WGCNA. Subsequently, these findings were cross-validated with the primary results in three ways: 1) probes of the chosen, significant modules were combined with the lists of pathways targets primarily considered to identify genes that overlapped between the results of TCGA and GEO data. Subsequently, logFC regarding the differential expression of the identified genes between LUSC and LUAD was calculated on TCGA and GEO data followed by a comparison of the expression trends referring to up- or downregulation (up: logFC > 0, down: logFC < 0) via Venn diagrams; 2) the analysis of ontological terms for each of WGCNA module was performed and compared with the list of initial annotation to identify commonly altered processes; and 3) dimensional grouping of LUSC and LUAD patients according to the resultant expression of genes that overlapped between each identified module and the list of pathway targets considered in the primary analysis by applying PCA with cancer subtype as qualitative supplementary variable.

Additionally, to confirm general trends regarding the gene expression in TCGA and GEO data, we extracted a total of 1000 genes with the highest logFC from our previously prepared TCGA data (20,502 genes) and cross-validated with Raponi’s and Ding’s datasets (13,513 genes). Moreover, we chose set of genes from the most significant WGCNA modules based on their differential expression profiles in LUSC and LUAD (logFC > 1.5) from the primary analysis and compared with patterns of expression with GEO data. Validation results together with tables (Supplementary Tables 2–4) and figures (Supplementary Figs. 5–9) are described in Supplementary Material.

Full lists of GSEA results and derived datasets as well as the source codes for R analyses are publicly available at GitHub repository (https://github.com/orzechmag/lungs).

## Supplementary information

Supplementary Information 1.

## Data Availability

Publicly available datasets were analysed in this study. These data can be found here: TCGA http://gdac.broadinstitute.org/; GEO https://www.ncbi.nlm.nih.gov/geo/.
