# Supplementary material for: Lung squamous cell carcinoma and lung adenocarcinoma differential gene expression regulation through pathways of Notch, Hedgehog, Wnt, and ErbB signalling
Source: Sci Rep. 2020 Dec 3;10:21128. doi: 10.1038/s41598-020-77284-8 (PMC7713208; doi:10.1038/s41598-020-77284-8)

**Lung Squamous Cell Carcinoma and Lung Adenocarcinoma differential gene expression regulation through pathways of Notch, Hedgehog, Wnt, and ErbB signalling**

Dorota Anusewicz ^1^†*, Magdalena Orzechowska ^1^† and Andrzej K. Bednarek ^1^

^1^ Department of Molecular Carcinogenesis, Medical University of Lodz, 90-752 Lodz, Poland

† These authors contributed equally to the work.

Supplementary material

**Data validation**

For study cross-validation, we performed analogous WGCNA analysis investigating profiles of expression differentiating LUSC and LUAD by employing two suitable microarray datasets of the size of 130 and 75 patients, respectively. Based on the conducted pipeline, we identified a total of 24 modules of co-expression. According to the module-trait relationship analysis, we focused on 8 relevant modules: black (r=0.79, p-value=2.21e-44), blue (r=0.95, p-value=2.43e-104), brown (r= -0.97, p-value=1.55e-128), greenyellow (r=0.86, p-value=3.48e-61), lightgreen (r=0.81, p-value=6.76e-50), magenta (r=0.86, p-value=3.7e-61), red (r=0.84, p-value=5.22e-57) and turquoise (r=0.998, p-value = 2.05e-253) (Supplementary Table 2). These modules were also the most promising regarding the GS across all identified as shown in Supplementary Figure 5. The profiles of expression exhibited by the genes in the particular modules that reflected differential biology of LUSC and LUAD are shown in the heatmaps (Supplementary Figure 6). Subsequently, each module was compared with the list of targets of Notch, Hh, ErbB and Wnt pathways, and restricted to the overlapping genes; logFC of median expression in LUSC and LUAD was calculated in TCGA and microarray data and finally compared in terms of the expression trend (expressed as up- or downregulation) via Venn diagrams (Table 1). In the majority, we observed remarkable matching trends of the expression in primary and validation results of WGCNA of approx. 50% or higher.

Regarding the biological mechanisms that differentially expressed genes clustered across WGCNA modules were involved in, we performed analogous gene ontology analysis according to KEGG canonical pathways and GO BP. Among the relevant modules, we identified multiple terms, which were generally associated with carcinogenesis, such as i.a. cell-cell signalling, regulation of intracellular signal transduction, regulation of cell differentiation, regulation of cell death, cellular homeostasis, cytoskeleton organisation, cell motility, cell cycle, regulation of cell population proliferation, regulation of response to stress, biological adhesion, tissue morphogenesis, regulation of MAPK cascade and MAPK signaling pathway, cell morphogenesis involved in differentiation, protein targeting to membrane, RNA processing, regulation of transferase activity, DNA repair, regulation of cell differentiation, apoptotic signalling pathway, cell cycle G1-S phase transition, double-strand break repair, mismatch repair, cell-cell signalling by Wnt, DNA integrity checkpoint, nucleotide excision repair, stem cell differentiation, and positive regulation of epithelial-to-mesenchymal transition that significantly confirmed the primary findings (Supplementary Table 3).

Formerly, we performed the analysis of spatial characteristics of LUSC and LUAD patients according to the resultant expression of the chosen downstream effectors of the pathways identified within the WGCNA modules. Indeed, we conducted a similar analysis by applying PCA to the genes that overlapped between significant modules within microarray data and primarily considered targets of the pathways. Notably, the resultant expression of the downstream effectors of Notch, Hh, ErbB and Wnt pathways that could be found among cross-validation modules partitioned LUSC and LUAD similarly to the previous findings. Importantly, LUSC patients were separated from LUAD along the PC1 and displayed a total variance of 68.8%, 66.9%, 66.3% and 66.5% for targets of Notch, Wnt, Hh and ErbB pathways, respectively, that overlapped between TCGA and microarray datasets (Supplementary Figure 7).

Additionally, to confirm general trends within the data, a total of 1000 genes with the highest logFC were chosen from our previously prepared TCGA data (20502 genes) and cross-validated with Raponi’s and Ding’s datasets (13513 genes). We found 487 common genes among TCGA and Gene Expression Omnibus (GEO) data, of which 338 genes were found to share common expression profiles (Supplementary Figure 8). Subsequently, we chose set of genes from the most significant WGCNA modules based on their differential expression profiles in LUSC and LUAD (logFC > 1.5). Of 146 common genes among TCGA and GEO datasets, 80 genes demonstrated the same pattern of expression (Supplementary Figure 9).

# Supplementary Tables

**Supplementary Table 1. Enrichment across WGCNA modules (MsigDB collection).**

| Module | Name | FDR |
| --- | --- | --- |
| Wnt |  |  |
| blue | Cell cycle | 8.71E-08 |
|  | DNA repair | 5.77E-10 |
|  | Response to DNA damage stimulus | 1.44E-09 |
|  | Response to stress | 1.95E-09 |
|  | Cytoskeleton organisation and biogenesis | 3.18E-08 |
|  | Cell proliferation | 2.04E-05 |
|  | Cell cycle checkpoint | 0.000112 |
|  | MAPK pathway | 0.000436 |
| green | Protein metabolic process | 0.00021 |
|  | Protein transport | 0.000218 |
|  | Intracellular transport | 0.000241 |
| ErbB |  |  |
| purple | Multicellular organismal development | 0.034967 |
|  | Intercellular junction assembly | 0.051269 |
|  | Protein metabolic process | 0.055238 |
| turquoise | Cell cycle | 3.79E-22 |
|  | DNA repair | 1.82E-17 |
|  | Response to DNA damage stimulus | 6.01E-15 |
|  | Response to stress | 2.07E-14 |
|  | Cell proliferation | 4.50E-12 |
|  | Base excision repair | 3.66E-14 |
|  | Mismatch repair | 1.12E-11 |
|  | Nucleotide excision repair | 2.78E-11 |
|  | Pyrimidine metabolism | 3.09E-08 |
|  | Cytoskeleton organization and biogenesis | 1.64E-07 |
|  | G1 to S transition of mitotic cell cycle | 0.000485 |
| Notch |  |  |
| brown | Apoptosis | 1.78E-05 |
|  | Programmed cell death | 1.80E-05 |
|  | Regulation of apoptosis | 0.000478 |
|  | Multicellular organismal development | 5.55E-07 |
|  | Cell development | 6.78E-07 |
|  | Regulation of metabolic process | 2.00E-05 |
|  | RNA metabolic process | 0.00072 |
| turquoise | RNA metabolic process | 4.19E-29 |
|  | Cellular protein metabolic process | 2.51E-25 |
|  | Cell cycle | 4.24E-25 |
|  | Protein metabolic process | 6.21E-25 |
|  | Cell proliferation | 2.42E-09 |
|  | DNA repair | 1.25E-10 |
|  | Response to stress | 2.01E-08 |
|  | Nucleotide excision repair | 3.11E-07 |
|  | Mismatch repair | 2.11E-06 |
|  | Cell cycle checkpoint | 5.99E-07 |
| yellow | Protein metabolic process | 8.58E-09 |
|  | Cell cycle | 1.05E-06 |
| Hedgehog |  |  |
| blue | Cell cycle | 4.31E-21 |
|  | Cell cycle phase | 1.47E-18 |
|  | Cellular protein metabolic process | 6.14E-13 |
|  | Response to stress | 1.97E-13 |
|  | DNA replication | 5.03E-13 |
|  | Response to DNA damage stimulus | 4.35E-07 |
|  | Cell cycle checkpoint | 2.38E-05 |
|  | Mismatch repair | 0.000346 |
|  | Nucleotide excision repair | 0.000242 |
|  | Cell proliferation | 3.52E-05 |
| brown | Cellular protein metabolic process | 1.70E-09 |
|  | Cell development | 4.69E-05 |
|  | Cytoskeleton organization and biogenesis | 0.000318 |
|  | Intracellular protein transport | 0.000418 |
| purple | Multicellular organismal development | 0.008337 |
|  | Intracellular signalling cascade | 0.013012 |
|  | Cell cycle | 0.015826 |
| red | DNA metabolic process | 0.000154 |
|  | RNA metabolic process | 0.000302 |
|  | Intracellular signalling cascade | 0.002466 |

Supplementary Table 2. Module-trait relationship of cross-validation WGCNA analysis involving microarray data.

| module | correlation coefficient (r) | p-value |
| --- | --- | --- |
| MEgrey60 | 0.247741585 | 0.000341727 |
| MEdarkturquoise | 0.287742291 | 2.87E-05 |
| MEdarkred | 0.265068589 | 0.000122569 |
| MEpurple | 0.357466041 | 1.43E-07 |
| MEdarkgreen | 0.294380121 | 1.83E-05 |
| MEcyan | 0.261327839 | 0.000153865 |
| MEtan | 0.753907834 | 6.79E-39 |
| MEmidnightblue | 0.639029086 | 6.35E-25 |
| MEred | 0.844690815 | 5.22E-57 |
| MEblack | 0.786597036 | 2.21E-44 |
| MEmagenta | 0.859766373 | 3.70E-61 |
| MEgreenyellow | 0.859857438 | 3.48E-61 |
| MEblue | 0.949728973 | 2.43E-104 |
| MEturquoise | 0.99833228 | 2.05E-253 |
| MElightgreen | 0.814528892 | 6.76E-50 |
| MEsalmon | 0.747151872 | 7.23E-38 |
| MElightyellow | -0.178050269 | 0.010644884 |
| MElightcyan | -0.639272956 | 6.02E-25 |
| MEbrown | -0.971274225 | 1.55E-128 |
| MEgreen | -0.719544263 | 5.51E-34 |
| MEpink | -0.728679166 | 3.23E-35 |
| MEgrey | 0.997984516 | 4.50E-245 |

**Supplementary Table 3. Enrichment across cross-validation WGCNA modules (MsigDB collection).**

| Module | Name | FDR |
| --- | --- | --- |
| blue | cell-cell signalling | 0.00287493 |
|  | regulation of intracellular signal transduction | 4.61E-38 |
|  | regulation of cell differentiation | 6.59E-22 |
|  | regulation of cell death | 0.028633676 |
|  | cellular homeostasis | 0.015774731 |
|  | cytoskeleton organisation | 0.021868396 |
|  | cell motility | 0.028797484 |
|  | cell cycle | 0.030533851 |
|  | regulation of cell population proliferation | 0.028109489 |
|  | regulation of response to stress | 0.0255377 |
|  | biological adhesion | 0.02334267 |
|  | tissue morphogenesis | 0.01097515 |
|  | cell morphogenesis involved in differentiation | 1.04E-08 |
| brown | protein targeting to membrane | 2.61E-47 |
|  | RNA processing | 1.36E-40 |
|  | DNA repair | 2.00E-20 |
|  | regulation of cell differentiation | 5.17E-18 |
|  | apoptotic signalling pathway | 2.09E-14 |
|  | cell cycle G1-S phase transition | 2.12E-09 |
|  | double-strand break repair | 2.43E-06 |
|  | mismatch repair | 3.49E-06 |
|  | cell-cell signalling by Wnt | 4.82E-06 |
|  | DNA integrity checkpoint | 5.32E-06 |
|  | nucleotide excision repair | 1.09E-05 |
|  | stem cell differentiation | 1.23E-05 |
| turquoise | epithelial-to-mesenchymal transition | 4.02E-14 |

**Supplementary Table 4**. The summarised results of comparison between primary and cross-validation WGCNA findings.

| module of cross-validation WGCNA [size] | Notch | | Hedgehog | | ErbB | | Wnt | |
| --- | --- | --- | --- | --- | --- | --- | --- | --- |
|  | overlap in trend* | overlap in the genes** | overlap in trend | overlap in the genes | overlap in trend | overlap in the genes | overlap in trend | overlap in the genes |
| black [33] | - | | - | | 4 | 17 | 4 | 7 |
| blue [396] | 9 | 18 | 12 | 29 | 10 | 29 | 47 | 50 |
| brown [424] | 3 | 5 | 11 | 28 | 20 | 162 | 72 | 98 |
| greenyellow [21] | - | | 1 | 1 | 5 | 21 | 0 | 9 |
| lightgreen [12] | - | | 1 | 2 | 1 | 3 | 2 | 6 |
| magenta [61] | 1 | 1 | 1 | 2 | 16 | 27 | 11 | 15 |
| red [33] | 0 | 1 | 0 | 1 | 9 | 14 | 4 | 12 |
| turquoise [3726] | 12 | 17 | 12 | 22 | 17 | 28 | 290 | 519 |

* defined as the total sum of genes that demonstrated the same trend in expression in the primary data (TCGA) and cross-validation studies (microarray datasets);

** defined as the total sum of genes that overlapped between the list of probes within primary data (TCGA) and cross-validation studies (microarray datasets)

**Supplementary Table 5. Clinical characteristics of LUSC and LUAD patients.**

| Clinical characteristic | LUAD | LUSC |
| --- | --- | --- |
| Total # of sample | 515 | 499 |
| Gender |  |  |
| female | 276 | 129 |
| male | 239 | 370 |
| Age at diagnosis |  |  |
| median/(range) | 66/(38-88) | 68/(39-90) |
| NA | 19 | 8 |
| Stage |  |  |
| I | 5 | 3 |
| IA | 131 | 90 |
| IB | 139 | 151 |
| II | 1 | 3 |
| IIA | 50 | 65 |
| IIB | 71 | 92 |
| III | 0 | 3 |
| IIIA | 73 | 63 |
| IIIB | 11 | 18 |
| IV | 26 | 7 |
| NA | 8 | 4 |
| Smoking history |  |  |
| lifelong non-smoker | 75 | 18 |
| current smoker | 119 | 133 |
| current reformed smoker for > 15 yrs | 135 | 83 |
| current reformed smoker for < or = 15 yrs | 168 | 249 |
| current reformed smoker, duration not specified | 4 | 5 |
| NA | 14 | 11 |
| Cancer status |  |  |
| tumor free | 342 | 337 |
| with tumor | 120 | 92 |
| NA | 53 | 70 |
| Vital status |  |  |
| alive | 389 | 341 |
| dead | 126 | 158 |

# Supplementary Figures


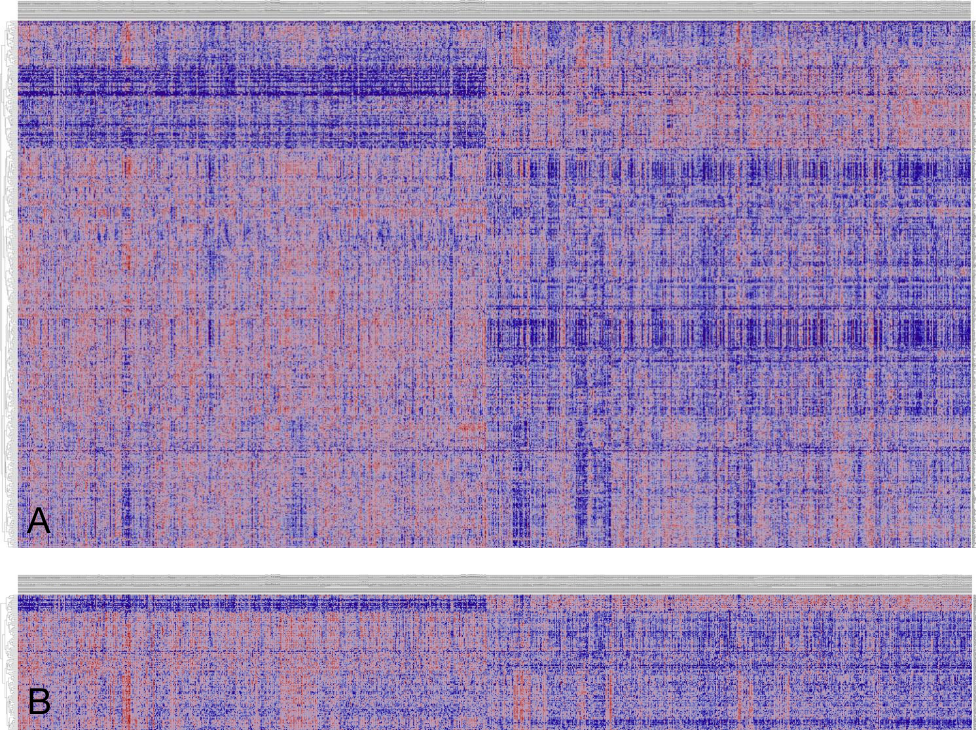


Supplementary Figure 1. Notch targets A) turquoise and B) yellow module expression.


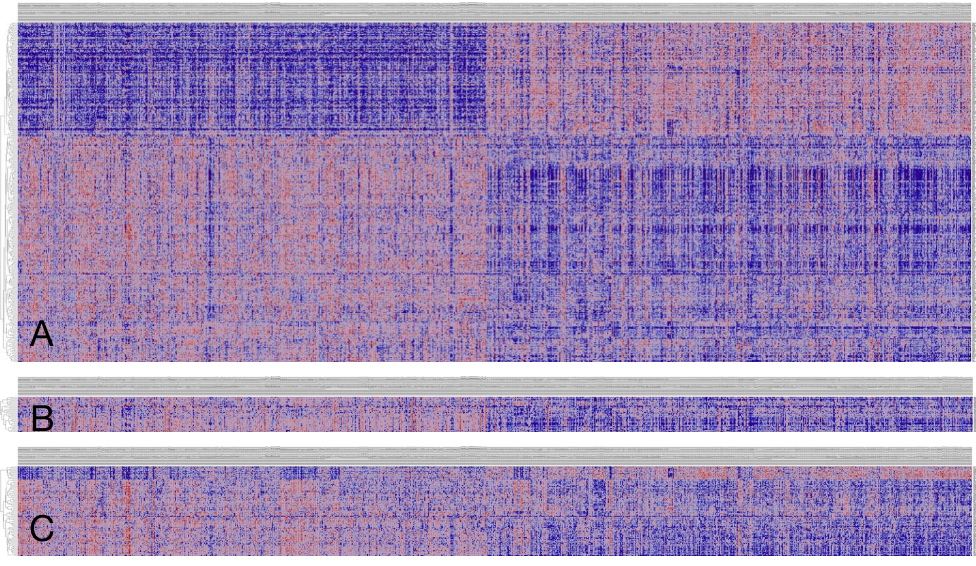


Supplementary Figure 2. Hh targets A) blue, B) purple and C) red module expression.


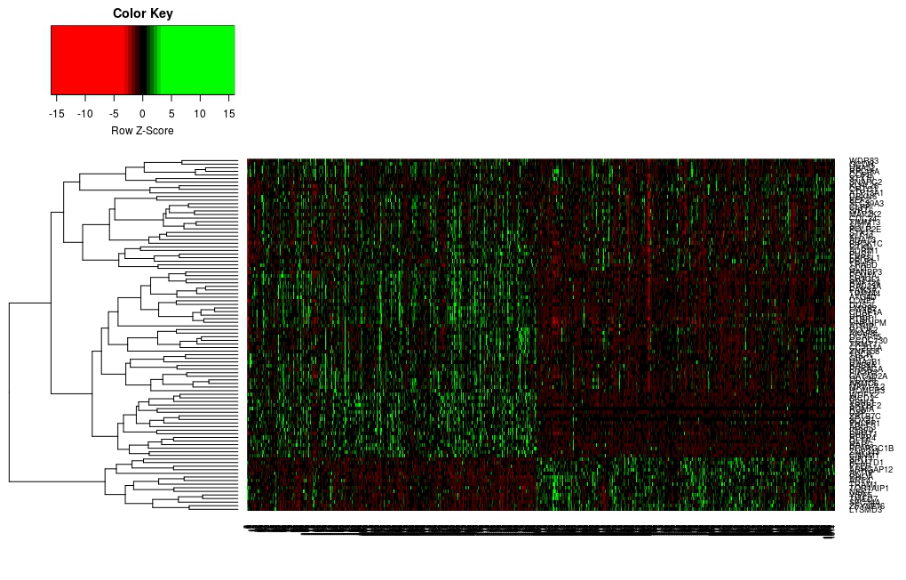


Supplementary Figure 3. Wnt targets green module expression.


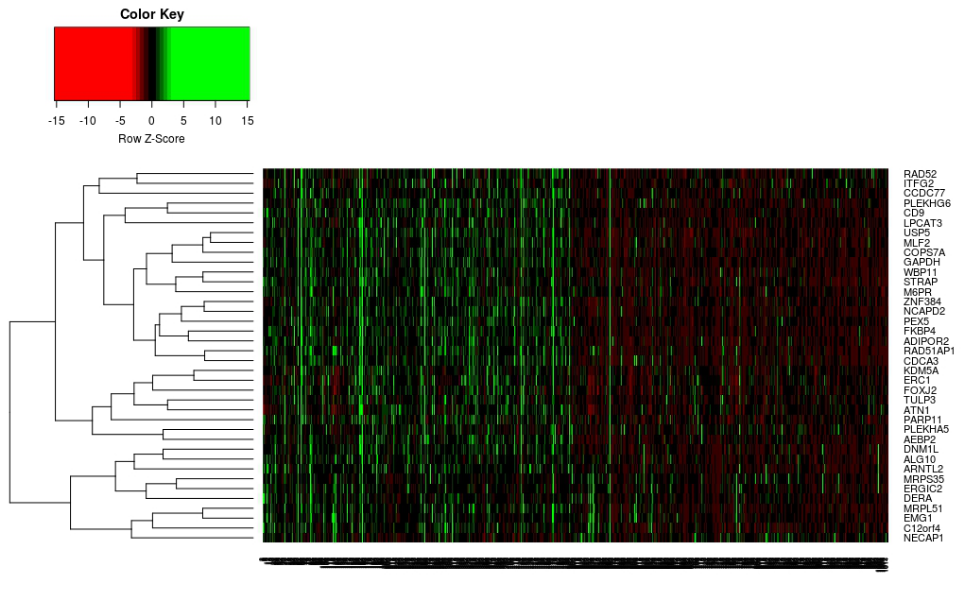


Supplementary Figure 4. ErbB targets purple module expression.


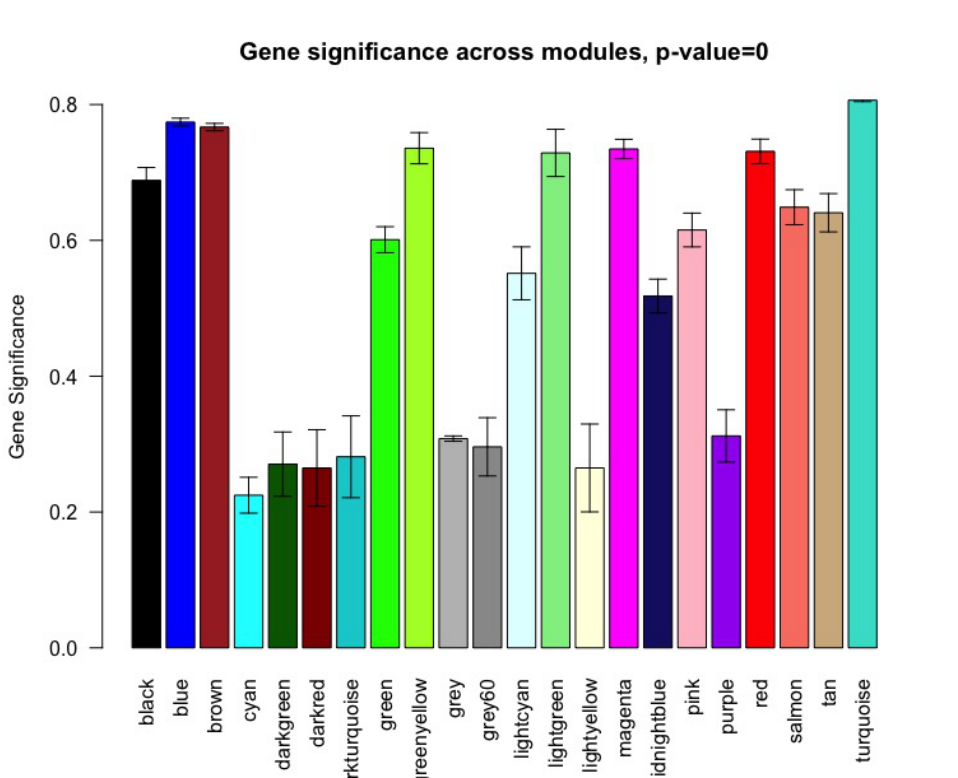


Supplementary Figure 5. Gene significance across the modules of cross-validation WGCNA analysis.


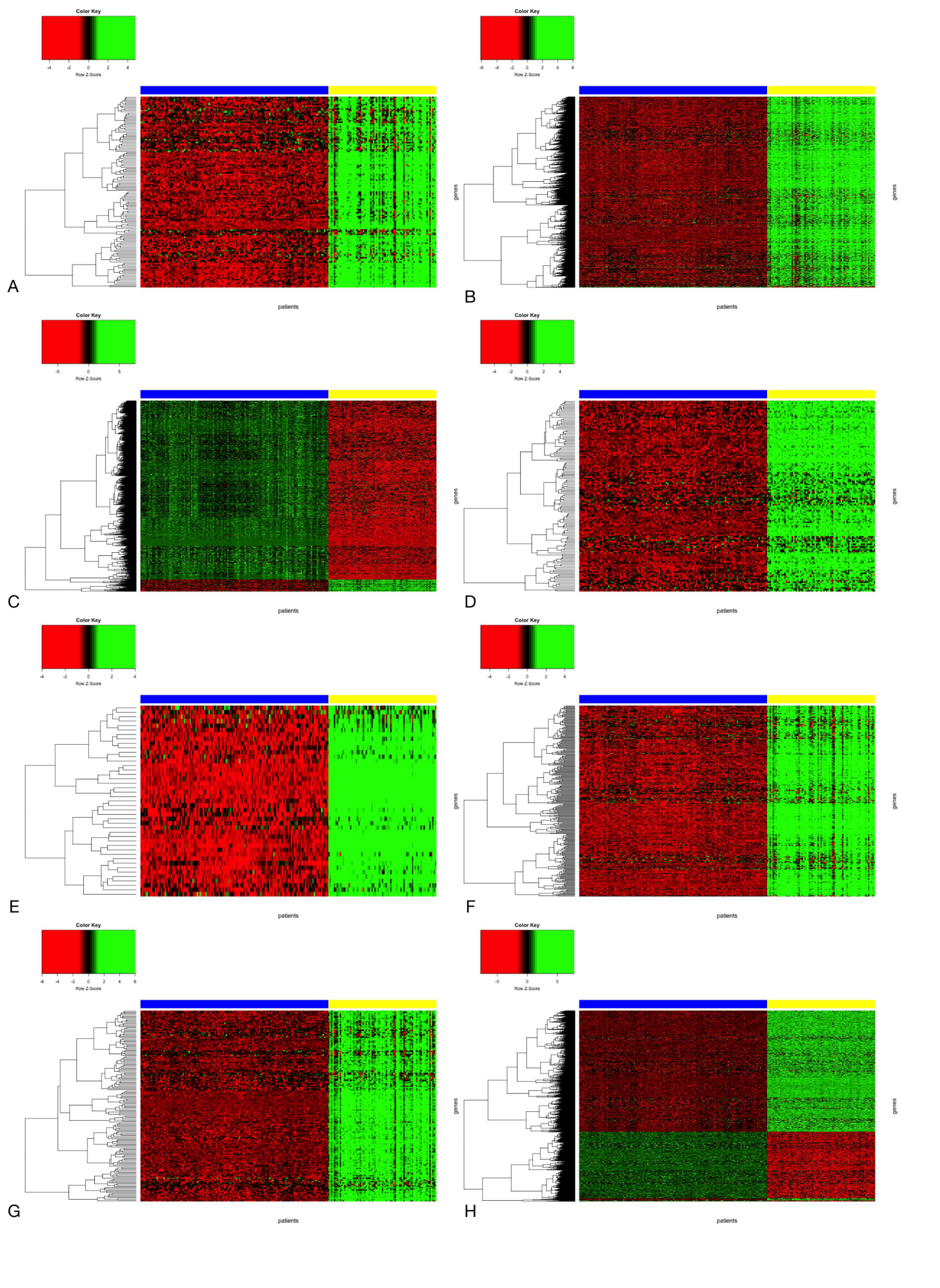


Supplementary Figure 6. Differential gene expression of the A) black, B) blue, C) brown, D) greenyellow, E) lightgreen, F) magenta, G) red and H) turquoise modules in LUSC (blue panel) and LUAD (yellow panel) microarray data.


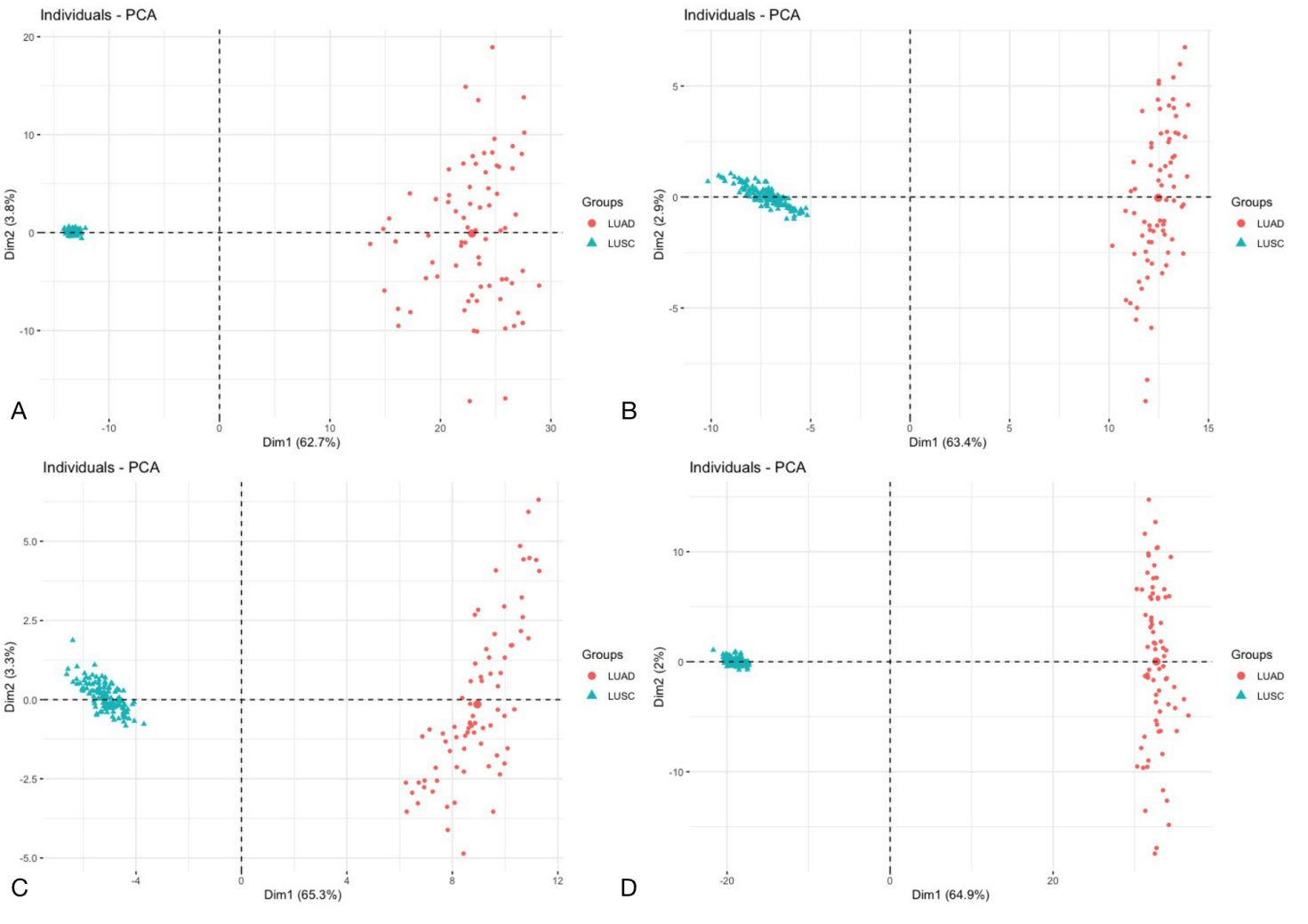


Supplementary Figure 7. Spatial partitioning of LUSC and LUAD patients based on the resultant expression of genes overlapping between cross-validation WGCNA analysis and A) ErbB, B) Hh, C) Notch and D) Wnt downstream targets.


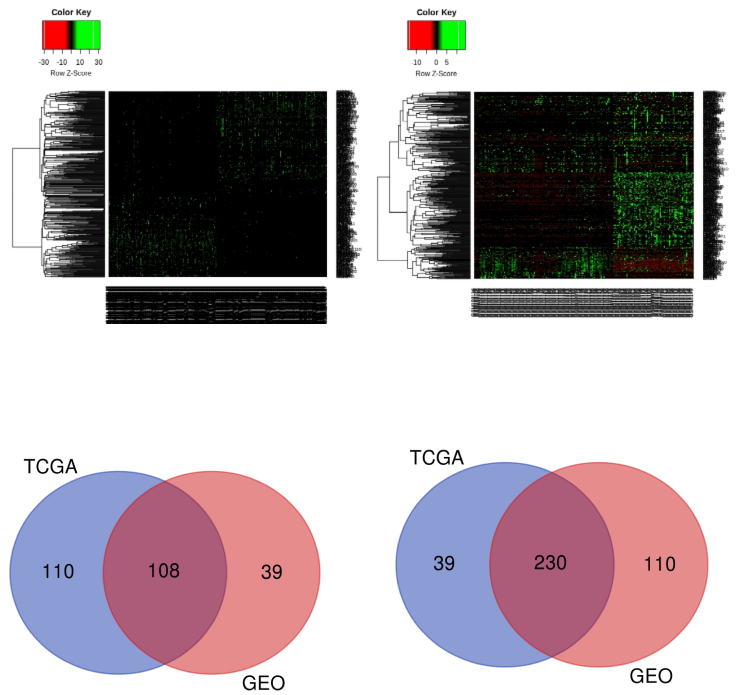


Supplementary Figure 8. Cross-validation of findings. Upper part: expression profiles of common genes of TCGA patients (left) and combined Ding and Raponi cohorts (right); bottom part: Venn diagrams showing genes of common and distinct expression profiles between TCGA and GEO cohorts presented on heatmaps.


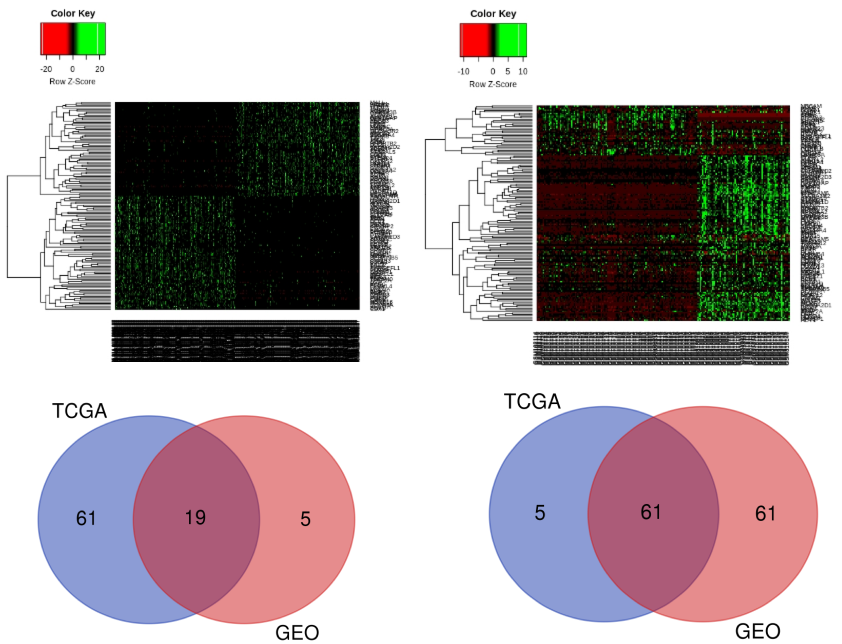


Supplementary Figure 9. Cross-validation of findings. Upper part: expression profiles of common genes of WGCNA modules of TCGA patients (left) and combined Ding and Raponi cohorts (right); bottom part: Venn diagrams showing genes of common and distinct expression profiles between TCGA and GEO presented on heatmaps.


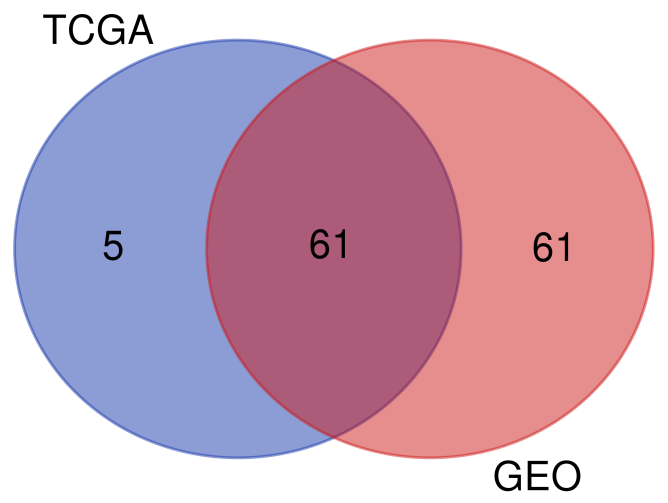

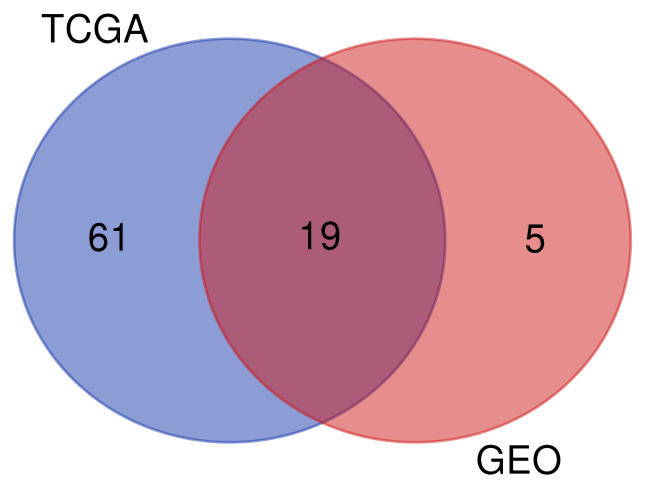

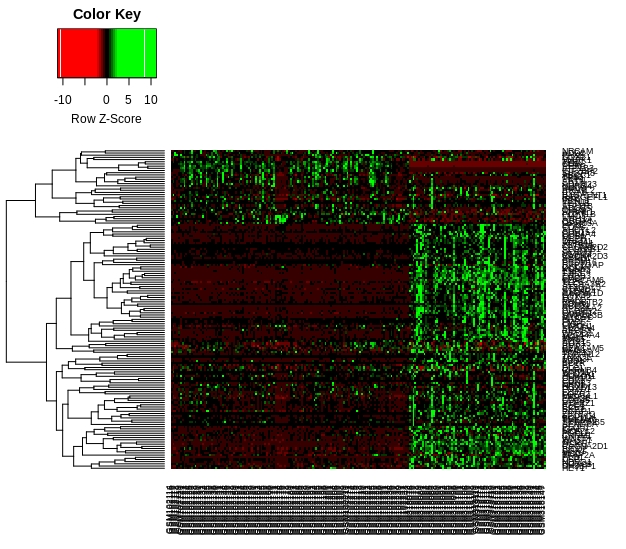

Supplement: Supplementary file 1 — Supplementary Information 1. [file 41598_2020_77284_MOESM1_ESM.docx]
